# Voxel-based morphometry and task functional magnetic resonance imaging in essential tremor: evidence for a disrupted brain network

**DOI:** 10.1038/s41598-020-69514-w

**Published:** 2020-09-15

**Authors:** Ilaria Boscolo Galazzo, Francesca Magrinelli, Francesca Benedetta Pizzini, Silvia Francesca Storti, Federica Agosta, Massimo Filippi, Angela Marotta, Giancarlo Mansueto, Gloria Menegaz, Michele Tinazzi

**Affiliations:** 1 Department of Computer Science, University of Verona, Strada Le Grazie 15, Ca’ Vignal 2, 37134 Verona, Italy; 2 Department of Neurosciences, Biomedicine and Movement Sciences, Neurology Section, University of Verona, Piazzale L.A. Scuro 10, 37134 Verona, Italy; 3 Department of Diagnostics and Pathology, University of Verona, Verona, Italy; 4 Neuroimaging Research Unit, Division of Neuroscience, Institute of Experimental Neurology, San Raffaele Scientific Institute, Vita-Salute San Raffaele University, Milan, Italy

**Keywords:** Neuroscience, Neurology, Movement disorders

## Abstract

The pathophysiology of essential tremor (ET) is controversial and might be further elucidated by advanced neuroimaging. Focusing on homogenous ET patients diagnosed according to the 2018 consensus criteria, this study aimed to: (1) investigate whether task functional MRI (fMRI) can identify networks of activated and deactivated brain areas, (2) characterize morphometric and functional modulations, relative to healthy controls (HC). Ten ET patients and ten HC underwent fMRI while performing two motor tasks with their upper limb: (1) maintaining a posture (both groups); (2) simulating tremor (HC only). Activations/deactivations were obtained from General Linear Model and compared across groups/tasks. Voxel-based morphometry and linear regressions between clinical and fMRI data were also performed. Few cerebellar clusters of gray matter loss were found in ET. Conversely, widespread fMRI alterations were shown. Tremor in ET (task 1) was associated with extensive deactivations mainly involving the cerebellum, sensory-motor cortex, and basal ganglia compared to both tasks in HC, and was negatively correlated with clinical tremor scales. Homogeneous ET patients demonstrated deactivation patterns during tasks triggering tremor, encompassing a network of cortical and subcortical regions. Our results point towards a marked cerebellar involvement in ET pathophysiology and the presence of an impaired cerebello-thalamo-cortical tremor network.

## Introduction

Essential tremor (ET) has recently been redefined as an isolated tremor syndrome characterized by bibrachial postural and/or kinetic tremor which has been present for at least 3 years and may affect other body parts (e.g. head, voice, lower limbs) but is not associated with other neurological signs (e.g., parkinsonism, dystonia, ataxia)^1^. This is a refinement of previous definitions of ET, in which the coexistence of additional neurological signs of uncertain significance, now labelled as “ET plus”, was accepted as ET^1^. Although the revised definition of ET has engendered debate among clinicians and researchers and is considered strict by some^2^, it has some advantages in research settings, where the selection of highly homogeneous cohorts is a pivotal requisite when designing mechanistic studies and clinical trials.

ET has an estimated prevalence of nearly 1% in the general population and 5% in people aged 65 and over, thus standing among the most common neurological disorders^3^. Despite its high prevalence, controversy exists on the pathophysiology of ET^4,5^. Several non-mutually exclusive pathomechanisms have been proposed so far^4^. First, ET might represent a neurodegenerative disease with prominent involvement of the cerebellar cortex in keeping with neuroradiological and pathological evidence^6–8^. Second, ET could be underpinned by a defective gamma-aminobutyric acid (GABA) neurotransmission which was demonstrated to partially colocalize with neurodegenerative changes, suggesting that both a reduction in GABA receptor density and alterations in GABA receptor functioning might contribute to lower the GABAergic tone^4^. Third, ET might be secondary to abnormal oscillatory activity within a tremor-generating network including the inferior olive, cerebellum, red nucleus, thalamus, and sensory-motor cortex^4,8^.

In this context, advanced magnetic resonance imaging (MRI) may provide novel insights to shed light on the pathophysiology of ET^5,7,9^.

From a morphological perspective, while post-mortem pathological studies have demonstrated the presence of ET-related changes in cerebellar structures^6^, the assessment of anatomical brain integrity in-vivo is more challenging and has been hampered by the quality of available data. Automatic techniques, such as voxel-based morphometry (VBM), can overcome these issues enabling to quantify local alterations in gray (GM) and white matter (WM) volume without a priori assumptions on tissue composition and to detect subtle alterations that elude visual inspection. However, previous studies relying on VBM to compare ET patients and healthy controls (HC) provided variable and inconsistent findings and call for further research^10–13^. Indeed, ET has been associated with either GM volume loss in bilateral cerebellar hemispheres alone or along with other brain regions^12,14–18^, or no differences in GM volumes^13,19–22^, or increased GM volumes of the bilateral cerebellum and right occipital fusiform gyrus^23,24^.

Considering this broad spectrum of results, recent studies on ET shifted from the assessment of brain anatomy only to its combination with brain activity information, generally retrieved from functional MRI (fMRI) based on blood-oxygenation-level-dependant (BOLD) contrast. BOLD signals arise from the complex interaction of neuronal, metabolic and vascular processes and therefore provide an indirect measure of neuronal activity^25^. Their acquisition can be obtained while the subject is resting in the scanner (rs-fMRI) or performing a task (task fMRI), conveying different information about the underlying brain activity. Rs-fMRI is a useful approach to explore brain functional organization and connectivity, but despite its undeniable relevance it cannot localize and lateralize spontaneous oscillations associated with different brain functions. Conversely, task fMRI enables to define brain areas which are involved in the execution of specific tasks by revealing perturbations of neuronal activity in terms of both increased (activation) and decreased (deactivation) BOLD signals^26,27^. Functional evidence currently available on ET mainly derives from rs-fMRI^28,29^, which demonstrated patterns of aberrant connectivity over several regions of the cerebello-thalamo-cortical network. Few studies have hitherto investigated ET using task fMRI, especially in combination with motor tasks, and their findings are largely not comparable. This may reflect inconsistencies in ET patient selection due to the longstanding absence of stringent diagnostic criteria and the lack of ET biomarkers, different MRI field strengths, acquisition schemes (in terms of both paradigms and tasks), and analysis methods^19,30–34^. In addition, previous task fMRI studies on ET focused only on BOLD activation patterns and did not explore deactivations, as frequently happened in the broader literature on fMRI. Negative BOLD responses were shown to reflect task-related decreases in neuronal activity compared to the spontaneous activity level at rest^35,36^, and have been reported in association with different paradigms, especially those requiring a greater task effort^37–39^. However, controversy still exists on their interpretation and whether deactivations reflect neuronal inhibition or hemodynamic compensatory mechanisms is still unclear, limiting the number of studies currently reporting their BOLD deactivation findings.

Overall, examining brain regions whose activity increases or decreases with tasks is highly important to understand how the brain works, and in the specific context of ET could help to unveil which regions are involved in tremor generation and propagation. Therefore, this study aimed to explore activation and deactivation brain mapping using task fMRI combined with a tremor-inducing motor task in a homogeneous cohort of ET patients diagnosed according to the most recent and stringent criteria^1^. These maps were also assessed with respect to physiological patterns derived from HC, aiming at identifying functionally relevant modulations, and linked to clinical data to assess whether a linear relationship between imaging and clinical manifestation exists. Finally, VBM was performed to quantify possible ET-related changes in terms of GM volume.

## Results

### Participants

Demographic and clinical characteristics of patients and controls are reported in Table 1. All patients with ET had bilateral arm tremor which was often mildly asymmetrical in amplitude on clinical assessment, being slightly worse in the dominant arm. Two patients showed associated mild head tremor in line with the inclusion criteria, four patients had also vocal tremor, and three patients had lower limb involvement. A family history of postural and/or kinetic tremor of the upper limbs was reported by seven ET patients. Age, gender, handedness, Beck Depression Inventory II score (BDI-II) and State-Trait Anxiety Inventory (STAI) score for trait anxiety (form Y2) were not significantly different among the two groups (*p* > 0.05).

**Table 1:** Demographic and clinical characteristics of patients with essential tremor (ET) and healthy controls (HC).

	ET (*n* = 10)	HC (*n* = 10)	*p* value^a^
**Demographic characteristics**			
Age (years)	69.4 ± 8.9	67.7 ± 7.8	0.579
Gender (M:F)	6:4	5:5	1.000
Handedness for writing (R:L)	10:0	10:0	1.000
**Characteristics of ET**			
Age of onset (years)	61.9 ± 11.6	NA	NA
Disease duration (years)	7.5 ± 3.4	NA	NA
*Body distribution*			
Upper limb	10	NA	NA
Head	2	NA	NA
Voice	4	NA	NA
Lower limb	3	NA	NA
Family history of ET	7	NA	NA
Response to alcohol	+ (2); − (4); CAN (4)	NA	NA
*Fahn–Tolosa–Marin TRS*			
TRS-A	6.9 ± 3.6	NA	NA
TRS-B	11.9 ± 5.6	NA	NA
TRS-C	5.1 ± 5.5	NA	NA
TRS total	23.9 ± 8.4	NA	NA
**Psychological assessment**			
BDI-II	10.7 ± 5.9	7.9 ± 4.3	0.280
STAI Y2	39.3 ± 8.9	36.9 ± 6.1	0.631

Results are provided as means ± standard deviations and absolute frequencies.

BDI-II, beck depression inventory II (score: 0–63; higher scores indicate more severe depressive symptoms); CAN, cannot answer; ET, patients with essential tremor; F, female; HC, healthy controls; L, left; M, male; *n*, units; NA, not applicable; R, right; STAI Y2, state-trait anxiety inventory form Y2 (score 20–80; higher scores indicate more severe trait anxiety); TRS, tremor rating scale (subscore A: 0–80, subscore B: 0–36, subscore C: 0–28, total score: 0–144; higher scores indicate more severe tremor; TRS scores were assessed while patients were off medication).

^a^Mann–Whitney U and Fisher’s Exact tests.

### VBM analysis

Significant volume loss was observed in ET patients compared to HC (*p* < 0.05, family-wise-error [FWE]-corrected) as revealed by the VBM analysis. The atrophy pattern predominantly involved the cerebellar structures, revealing four different clusters. The first cluster (*p*_*FWE*_ = 0.013, 1488 voxels), centered in the left lobule VIIIa, encompassed a broad area across vermis VIIIa, left lobule VIIb and Crus II. The second one (*p*_*FWE*_ = 0.004, 1050 voxels) involved vermis VIIIa, right Crus II, lobule VIIIa, and lobule VIIb, where the peak voxel was found. The left and right Crus I areas also showed a statistically significant volume reduction in patients relative to HC (*p*_*FWE*_ = 0.028, 135 voxels and* p*_*FWE*_ = 0.036, 43 voxels, respectively). In particular, the latter cluster also spread to the right lobule VI. Finally, a single cluster of cortical atrophy of the right occipital fusiform gyrus was found, although being of a limited extent (*p*_*FWE*_ = 0.043, 14 voxels; Figure S1).

**Figure 1:**
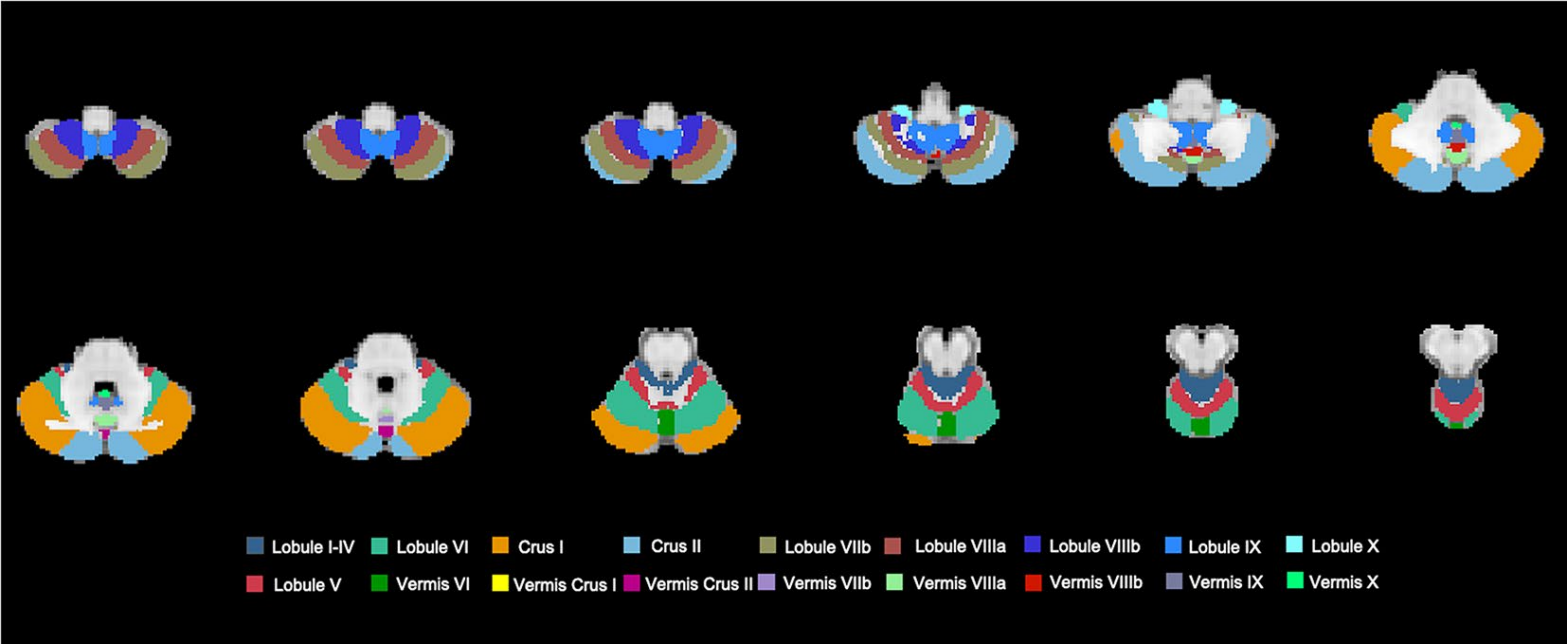
Schematic of the cerebellum. Representation of the major anatomical subdivisions of the cerebellum according to FSL probabilistic cerebellar atlas (non-linearly registered to MNI space).

A schematic of the cerebellum, following the FSL probabilistic cerebellar atlas, is shown in Fig. 1 for ease of interpretation.

### Task fMRI: general linear model (GLM) and statistical analyses

All participants completed the experimental paradigms and the analysis of the head motion parameters reported no statistically significant changes in terms of movement across groups (task 1 ET vs HC; task 1 in ET vs task 2 in HC) or tasks (task 1 vs task 2 in HC). Indeed, Supplementary Materials Table S1 shows that the means of the time course for the six movement parameters are limited with no significant group/task differences (*p* > 0.05).

Group GLM results are summarized in Supplementary Materials Tables S2–S3, while the statistical comparisons for (de)activations are reported in Tables 2–3. In particular, each significant cluster identified by the group/statistical analyses (*p* < 0.05, FWE-corrected) is reported with its name, the number of voxels, the value of the maximum *z*-statistic within the cluster, and the location of the maximum intensity voxel, given as X/Y/Z coordinate values in MNI (Montreal Neurological Institute) standard space coordinates (mm).

**Table 2:** Between-group comparison of activated and deactivated brain regions during task 1 in patients with essential tremor (ET) and healthy controls (HC).

ROI	Voxels	MAX *Z*-statistic	MAX X (mm)	MAX Y (mm)	MAX Z (mm)
**ACTIVATIONS**					
***TASK 1—ET > HC***					
FP.l	717	8.26	66	84	39
PRG.r/MFG.r	326	7.75	19	66	48
FP.r	261	9	32	88	31
Caud.r	111	6.19	36	75	34
PRG.l	101	8.47	61	58	60
***TASK 1—ET < HC***					
CER.r	484	7.52	42	40	32
HIPP.r	201	7.17	31	59	26
POG.l	105	7.4	61	45	64
THL.l/Caud.l	104	5.45	51	56	45
SMA	101	6.16	48	51	61
**DEACTIVATIONS**					
***TASK 1—ET > HC***					
CER.l	8593	− 15.4	48	19	18
PRG.r/POG.r/LOC.r	3776	− 9.73	28	39	55
PRG.l/POG.l/LOC.l/SMA	3279	− 10.3	67	32	51
CER.r	1588	− 11.4	35	18	17
PAC	342	− 6.84	48	75	59
CGp.l	281	− 8.66	47	41	45
FP.r	252	− 6.73	25	88	49
AMY.r	178	− 7.36	32	60	28
THL.r/Caud.r/THL.l/Caud.l	122	− 6.4	36	53	42
***TASK 1—ET < HC***					
FP.l	100	− 7.9	61	83	52

Voxels, number of voxels in each significant cluster; MAX *Z-*statistic, value of the maximum *z-*statistic within the cluster; MAX X/Y/Z (mm), location of the maximum intensity voxel, given as spatial coordinate values in standard space (mm). For all clusters, the corresponding *p* values are FWE-corrected and < 0.05. For ease of reading, a cluster size of at least 100 voxels has been chosen for reporting the significant clusters resulting from the statistical comparisons.

AMY, amygdala; Caud, caudate; CER, cerebellum; CGp, posterior cingulate gyrus; ET, patients with essential tremor; FP, frontal pole; FWE, family-wise error; HC, healthy controls; HIPP, hippocampus; l, left; LOC, lateral occipital cortex; MFG, middle frontal gyrus; PAC, paracingulate gyrus; POG, postcentral gyrus; PRG, precentral gyrus; r, right; ROI, region of interest; SMA, supplementary motor areas; THL, thalamus.

**Table 3:** Between-group comparison of activated and deactivated brain regions during task 1 in patients with essential tremor (ET) and during task 2 in healthy controls (HC).

ROI	Voxels	MAX *Z-*statistic	MAX X (mm)	MAX Y (mm)	MAX Z (mm)
**ACTIVATIONS**					
***TASK 1 ET > TASK 2 HC***					
FP.l	537	8.29	60	91	43
TP.r/MTG.r	419	6.4	19	60	23
FP.r/MFG.r	257	7.55	32	88	31
PAC	219	5.49	51	85	35
PRG.l/MFG.l	101	7.68	61	60	62
***TASK 1 ET < TASK 2 HC***					
CER.r/CER.l	6950	23.3	35	36	24
PRG.l/POG.l/LOC.l (sup)	4339	23.5	66	49	59
SMA	1569	10.8	43	54	64
PRG.r/POG.r/INS.r	975	9.88	13	65	45
LOC.r (sup)/SGp.r	529	10	22	34	63
Cau.r/THL.r	347	8.14	37	59	41
Cau.l/THL.l	295	7.92	48	57	41
Put.l/INS.l	140	7.85	63	62	37
FOC.r/IFG.r	245	7.97	28	78	38
FP.r	226	9.59	25	81	44
CGa.r/CGa.l	121	7.52	45	72	47
CN/CGp.r/CGp.l	105	7.06	45	21	45
**DEACTIVATIONS**					
***TASK 1 ET > TASK 2 HC***					
CER.r/CER.l	8734	− 17.9	55	33	23
POG.l/PRG.l/SMA/ LOC.l (sup/inf)/THL.l/ Put.l/Caud.l/OP.l	6053	− 22.7	67	49	59
POG.r/PRG.r/LOC.r (sup)/THL.r/ Caud.r	2014	− 13	33	45	70
INS.l	245	− 7.62	63	61	37
OP.r	188	− 8.67	34	19	45
LOC.r (inf)/TO3.r	149	− 11.4	24	40	30
INS.r/AMY.r	146	− 7.93	26	63	34
CGp.r/CGp.l	142	− 8.15	47	41	44
CGa.r/CGa.l	104	− 9.53	42	74	58
***TASK 1 ET < TASK 2 HC***					
LOC.l (inf)/OP.l	177	− 13	49	19	34
OP.r	109	− 5.93	28	18	45

Voxels, number of voxels in each significant cluster; MAX *Z-*statistic, value of the maximum *z-*statistic within the cluster; MAX X/Y/Z (mm), location of the maximum intensity voxel, given as spatial coordinate values in standard space (mm). For all clusters, the corresponding *p* values are FWE-corrected and < 0.05. For ease of reading, a cluster size of at least 100 voxels has been chosen for reporting the significant clusters derived from the statistical comparisons.

AMY, amygdala; Caud, caudate; CER, cerebellum; CGa, anterior cingulate gyrus; CGp, posterior cingulate gyrus; CN, cuneal cortex; ET, patients with essential tremor; FOC, frontal orbital cortex; FP, frontal pole; FWE, family-wise error; HC, healthy controls; IFG, inferior frontal gyrus; inf, inferior; INS, insular cortex; l, left; LOC, lateral occipital cortex; MFG, middle frontal gyrus; MTG, middle temporal gyrus; OP, occipital pole; PAC, paracingulate gyrus; POG, postcentral gyrus; PRG, precentral gyrus; Put, putamen; r, right; ROI, region of interest; SGp, posterior supramarginal gyrus; sup, superior; SMA, supplementary motor areas; THL, thalamus; TO3, inferior temporal gyrus, temporooccipital part.

### GLM group analysis

**Figure 2:**
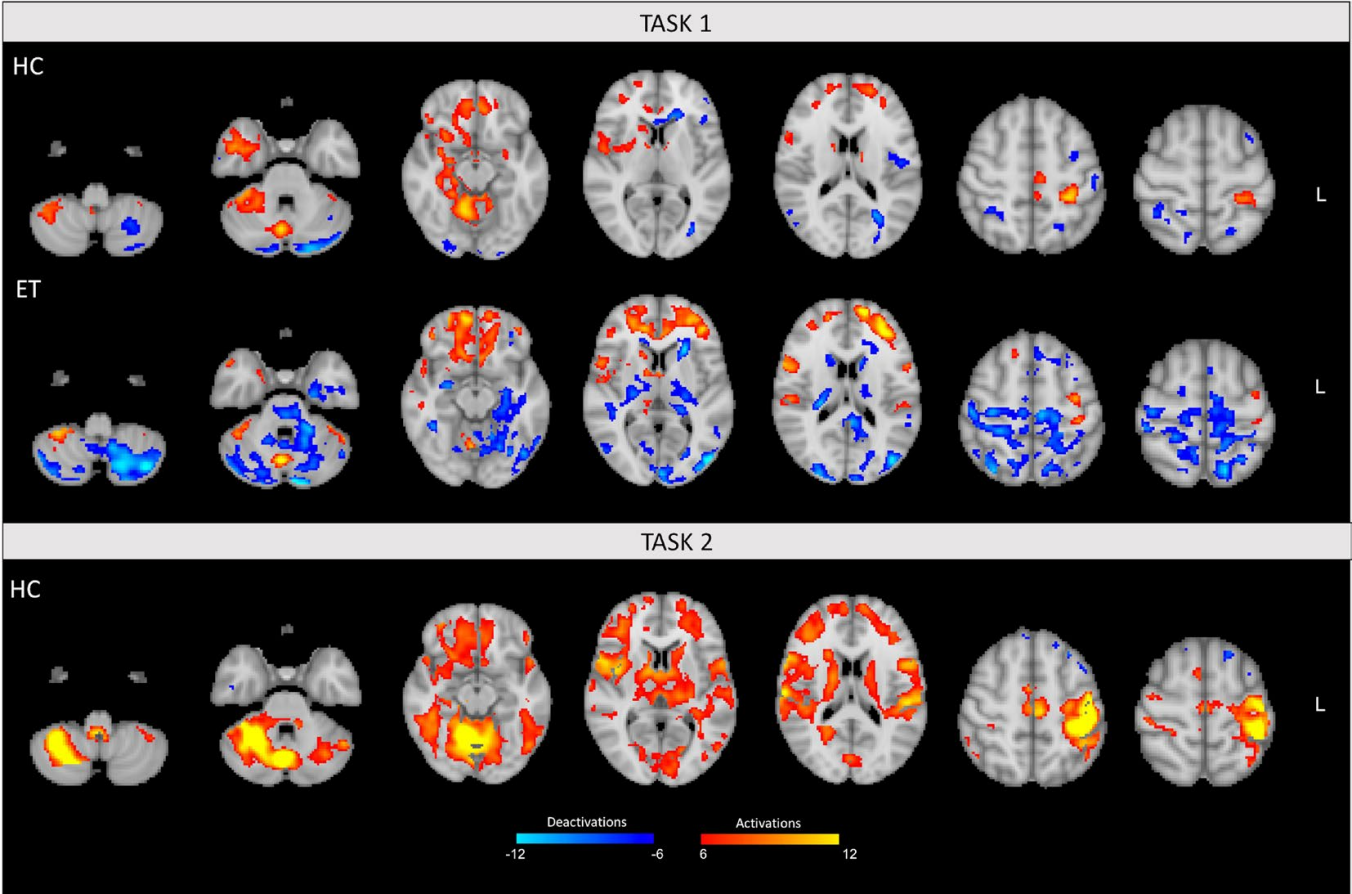
Activation and deactivation maps. Fixed-effects group analysis results for task 1 (patients with essential tremor [ET] and healthy controls [HC]) and task 2 (HC only). Statistical maps are thresholded by using clusters determined by Z > 6 (activations) and Z < − 6 (deactivations) with a (corrected) cluster significance threshold of *p* < 0.05.

*Control subjects*. Both activations and deactivations could be observed in the HC group. In particular, in task 1 the outstretching of the right arm was associated with significant bilateral activations of the cerebellum (mainly homolaterally over lobules I–VI, VIIIa, and Crus I), frontal and subcortical areas, such as caudate nucleus, putamen, thalamus. Activations limited to the contralateral cortical motor areas were also found (pre/postcentral gyri), and a homolateral activation of the temporal areas and insula was detected (Figs. 2 and S2, Table S2). Considering the deactivations associated with this task, a significant bilateral involvement of frontal and occipital areas plus cerebellum (mainly contralaterally over Crus I/II) could be detected in task 1 (Figs. 2 and S2, Table S3).

In task 2, the voluntary oscillation of the right wrist induced significant and extensive bilateral activations of the cerebellum (lobules V–VI and Crus I), cortical motor areas (pre/postcentral gyri, supplementary motor areas), and subcortical regions (thalamus and caudate nucleus). Predominantly contralateral activation of frontal areas, and homolateral activation of the insula were also detected in this condition (Figs. 2 and S2, Table S2). When considering the deactivation, limited albeit significant deactivated clusters could be observed mainly over the contralateral frontal and posterior areas (as posterior cingulate gyrus) (Figs. 2 and S2, Table S3).

*Patients*. In the ET group, postural tremor on extension of the right arm was mainly associated with extended bilateral activations of the frontal pole and caudate, contralateral activation of the pre/postcentral gyri, and with homolateral activation of frontal areas (precentral and middle frontal gyri), cerebellum (lobules V, VI, VIIIa, VIIIb, Crus I), temporal areas, and parietal opercular cortex (v, Table S2). In addition, a massive and unique cluster of deactivations was found, encompassing in particular the main motor areas, posterior areas and subcortical structures. Finally, an extended deactivation over the cerebellum was mainly detected in bilateral Crus I/II, left lobule VI, and left VIIb (Figs. 2 and S2, Table S3, Figure S1).

### Statistical analysis: between-group within-task comparison

**Figure 3:**
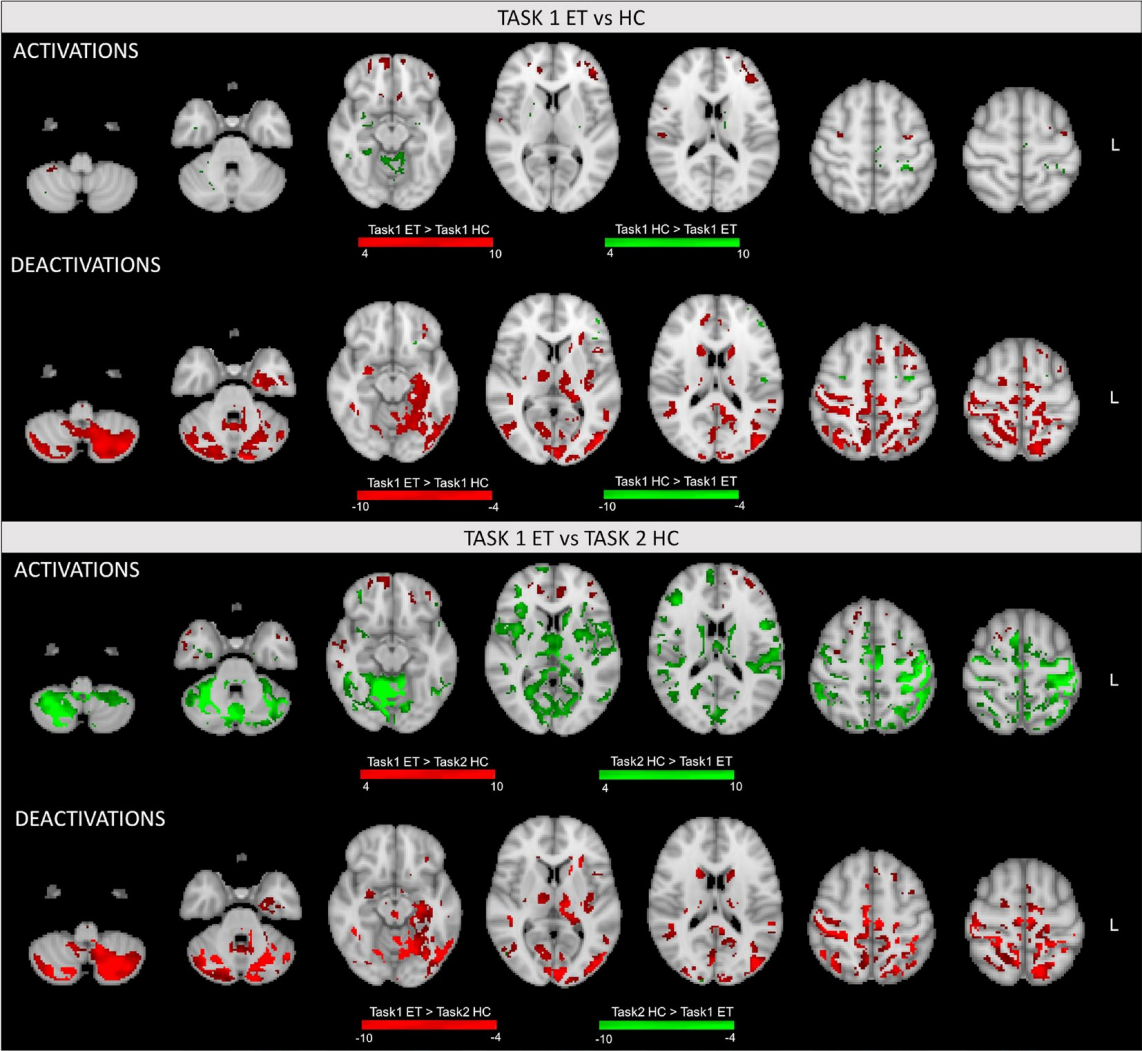
Statistical comparisons for activations and deactivations. Brain areas statistically different across groups (patients with essential tremor [ET] and healthy controls [HC]) and tasks are illustrated in figure and color-coded by statistical significance. Activations and deactivations were compared separately in the statistical analyses, and significant clusters were determined by Z > 4 (activations) and Z < − 4 (deactivations) with a (corrected) cluster significance threshold of *p* < 0.05. Top: between-group comparison (task 1 ET vs HC). Bottom: between-group between-task comparison (task 1 ET vs task 2 HC).

When comparing the areas elicited by task 1 in the two groups, we found few clusters of significantly higher activation in ET patients compared to HC, mainly over the bilateral frontal pole and precentral gyrus. Conversely, widespread significantly stronger deactivations were observed, especially in the bilateral Crus I/II (with contralateral predominance), contralateral lobules V, VI and VIIb, pre/postcentral gyri, supplementary motor areas and subcortical structures (as caudate and thalamus) (Figs. 3 and S3, Table 2).

On the other hand, HC showed few clusters of stronger activations in the homolateral cerebellum (mainly lobules I–V), hippocampus, contralateral postcentral gyrus, supplementary motor areas, thalamus, and caudate nucleus as well as few voxels of significantly higher deactivation mainly in the contralateral frontal pole (Figs. 3 and S3, Table 2).

### Statistical analysis: between-group between-task comparison

The between-group between-task comparison (task 1 in ET vs task 2 in HC) demonstrated few areas of significantly higher activations in patients compared to HC mimicking the tremor mainly over frontal and temporal areas bilaterally. Conversely, ET patients showed a large cluster of significantly higher deactivations spreading across the two cerebellar hemispheres (8734 voxels). In particular, as shown in Figs. 3 and S4, this cerebellar cluster extends mainly over the bilateral Crus I and Crus II, and contralateral lobules I–VI, VIIb, and IX. ET patients also showed a large cluster of significantly higher deactivation (~ 6000 voxels) mainly encompassing contralateral pre/postcentral gyri, lateral occipital cortex, thalamus, caudate nucleus, and putamen. A subset of these regions in the homolateral cerebral hemisphere were also more strongly deactivated in ET patients, resulting in a cluster of 2014 voxels.

The simulated tremor in the HC group was mainly associated with a stronger activation of the motor circuit compared to patients, in particular over the bilateral cerebellum (lobule VI and Crus I), homolateral lobules I–V, VIIb, VIIIa and Crus II, pre/postcentral gyri, and supplementary motor areas. Finally, few small clusters of significantly higher deactivations were found over the occipital areas for task 2 in HC compared to task 1 in ET (Fig. 3, Table 3).

### Statistical analysis: within-group between-task results

For the within-group between-task comparison in HC, activated voxels were significantly higher in the homolateral temporal areas, paracingulate gyrus, and in the contralateral frontal pole during extension of the right arm (task 1) compared with voluntary oscillation of the right wrist (task 2). In addition, we found significantly higher deactivations in contralateral postcentral gyrus, and lateral occipital cortex (Figures S5–S6, Table S4). A single cluster of deactivation limited to the contralateral cerebellar hemisphere was also found for task 1 > task 2 statistical comparison (134 voxels), encompassing lobules VIIb, VIIIa, and Crus I/II which are engaged in sensorimotor and cognitive processes.

Conversely, activations induced by task 2 were significantly higher than task 1 in several areas of the motor circuit, revealing extensive clusters over contralateral pre/postcentral gyri and supplementary motor areas. Bilateral, albeit predominantly homolateral, cerebellar changes were also present, mainly encompassing bilateral lobule VI and corresponding vermis, along with homolateral lobules V, VIIb, VIIIa, and Crus I. Contralateral lobule V and Crus I were also more strongly activated during task 2 compared to task 1. These cerebellar regions are involved in motor, sensorimotor, and cognitive processing. Significantly higher deactivations over the bilateral temporal pole and posterior/frontal areas were finally found (Figures S5–S6, Table S4).

### Task fMRI linear regression analysis with Fahn–Tolosa–Marin tremor rating scale (TRS) scores

**Figure 4:**
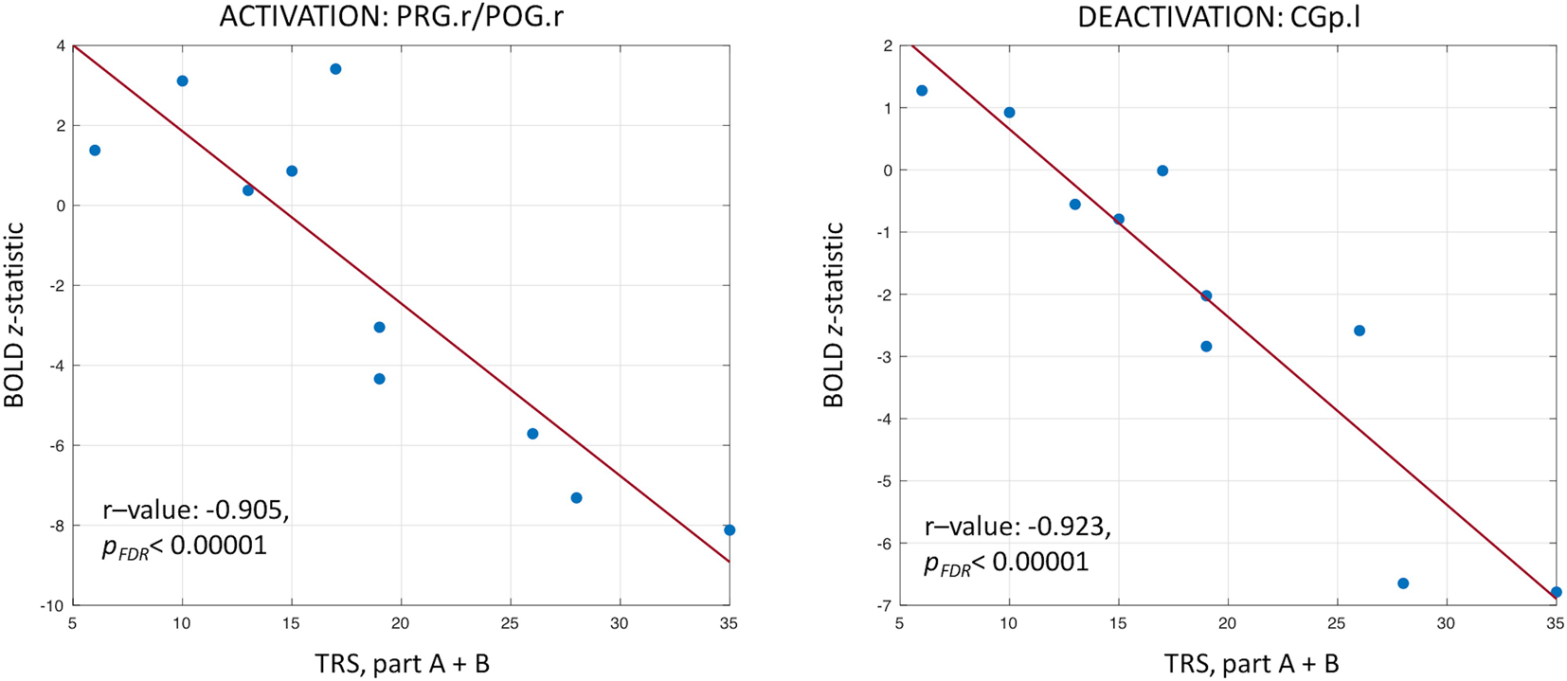
Linear regression analysis. Regression results for two representative regions of interest are reported, one resulting as activated in the general linear model analysis for task 1 in patients with essential tremor (right pre/postcentral gyrus [PRG.r/POG.r]) and one as deactivated (left posterior cingulate gyrus [CGp.l]). Individual BOLD results, expressed as *z-*statistic values, were linearly regressed against the Fahn–Tolosa–Marin Tremor Rating Scale (TRS), part A + B. The correlation value (r-value) and the corresponding false-discovery rate (FDR)-corrected *p*-value (*p*_*FDR*_) are also reported.

Detailed results of the regression analyses are reported in Supplementary Table S5. In particular, the clusters resulting from the voxelwise linear regression, subsequently masked with the BOLD (de)activations from the group analysis on ET patients, are indicated with their name, location of the maximum intensity voxel (X/Y/Z coordinates in MNI standard space), voxel size, correlation (r-value) and slope regression coefficient (beta value) together with their associated false-discovery-rate (FDR)-corrected *p*-values. The results for two representative regions (one activated and one deactivated as revealed by the group GLM analysis for task 1) are shown in Fig. 4, reporting the negative relationship between the BOLD *z*-statistic values and the TRS, part A + B.

A negative correlation between the TRS scores and BOLD values was found in all cases. Of note, only clusters with r-value < − 0.8 are here reported (with *p*_*FDR*_ < 0.001 in all cases), while all the significant clusters are detailed in Table S5. A negative strong correlation with the TRS, part A was observed in a number of activated areas, including the right inferior/middle frontal gyri (r = − 0.923), anterior cingulate cortex (r = − 0.879), left pre/postcentral gyri (r = − 0.898), and in few deactivated areas, encompassing part of the left pre/postcentral gyri (r = − 0.891) and the right posterior cingulate gyrus/precentral gyrus (r = − 0.861).

The TRS, part A + B was negatively correlated with similar areas as before. For the activated areas, a negative linear relationship was found for several frontal areas (left and right anterior cingulate cortex/superior frontal gyrus/frontal pole with r = − 0.918 and r = − 0.859, respectively; right inferior/middle frontal gyri with r = − 0.892) and motor areas (right and left pre/postcentral gyri with r = − 0.905 and r = − 0.803, respectively). For deactivation, a high correlation was found over the left posterior cingulate gyrus (r = − 0.923).

Finally, a negative correlation existed between the TRS total score and a number of activated areas, including the right inferior/middle frontal gyrus/precentral gyrus (r = − 0.876) and the left central opercular cortex (r = − 0.840). A negative correlation with the TRS total score was also observed in some deactivated areas, including the left posterior cingulate gyrus (r = − 0.829).

## Discussion

This study explored in-vivo morphological and functional brain modulations associated with ET in a homogeneous cohort of patients diagnosed according to the 2018 consensus criteria^1^ by using VBM and task fMRI. VBM showed few clusters of focal GM atrophy in both cerebellar hemispheres with respect to HC, but these regions did not overlap with activated/deactivated brain areas on fMRI. In addition, ET patients displayed statistically different activation and deactivation mapping when tremor triggered by maintaining a posture with their right arm (task 1) was compared with both the same task and the simulation of tremor (task 2) in HC. As novel finding, ET patients revealed a widespread deactivation circuit associated with the postural component of their tremor, encompassing several motor areas (e.g., pre/postcentral gyri, supplementary motor areas and cerebellum) and subcortical structures (e.g., thalamus and putamen). Finally, a linear negative relationship between imaging (BOLD *z-*statistics) and clinical variables (TRS scores) was found over several areas, reaching high significances.

From a morphological perspective, a significant GM volume loss (atrophy) was detected in posterior lobules of both cerebellar hemispheres in ET patients compared with HC, although not overlapping with the activated/deactivated cerebellar clusters shown in ET. These findings revealed the presence of a quite symmetric pattern of atrophy involving mainly, albeit not exclusively, cerebellar regions engaged in non-motor processing (sensorimotor, attention/executive functions, default-mode)^40,41^. A much smaller cluster of significant GM atrophy was localized to the right occipital fusiform gyrus in the ET group.

Several studies have hitherto investigated ET patients through VBM providing variable and inconsistent results^10–13^. In some agreement with our findings, quite a few studies reported GM volume loss in bilateral cerebellar hemispheres either isolated or associated with GM density reduction in other brain regions, such as occipital lobes^12,14–17^. Moreover, Bhalsing et al. detected GM volume reduction covering the anterior and posterior lobules of the cerebellum bilaterally in ET associated with cognitive impairment^18^. On the other hand, several studies did not show any differences in GM cerebral and cerebellar volumes between ET patients and HC^13,19–22^, whereas others reported increased GM volumes of the bilateral cerebellum^23,24^ and right occipital fusiform gyrus^24^ in ET patients. Discrepancies among studies might be attributable to patient selection reflecting, among others, less stringent diagnostic criteria for ET in the past, as well as to different MRI field strengths and heterogeneous anatomical sequences acquired in the populations^11^. In order to minimize all these possible confounding factors and derive reliable information, in our age-matched case–control study we enrolled a homogenous cohort of ET patients, relied on high-resolution volumetric anatomical images at 3 T and analyzed the data using state-of-the-art methods with a very stringent multiple comparison correction. All these elements allowed us to provide clear evidence in favor of a modulation of GM volume in specific areas of the cerebellum, as the analysis revealed nearly isolated clusters of cerebellar GM density reduction in our ET population which may be interpreted as regional atrophy in the context of a neurodegenerative disorder. Our VBM results might add some evidence to post-mortem findings of pathological changes in the cerebellar cortex, including reduction of Purkinje cell number, in several series of ET patients^6,42,43^, thus supporting the pathophysiological hypothesis that ET is a neurodegenerative disorder centered in the cerebellum.

From a functional perspective, main findings from our study are that: (1) postural tremor visible in ET patients with the right arm outstretched (task 1) was associated with significantly higher activation of bilateral frontal areas compared to task 1 and simulated tremor with the right arm outstretched (task 2) in HC; (2) ET patients during task 1 showed significantly higher deactivation of cerebellar regions engaged in both motor and non-motor processing, sensory-motor cortex, and basal ganglia bilaterally compared to task 1 in HC; (3) postural tremor in ET patients was associated with an increased deactivation of the cerebellum (again in lobules associated with motor and non-motor processes), sensory-motor cortex, lateral occipital cortex, thalamus, and caudate nucleus bilaterally compared to task 2 in HC. The consistency of these findings was supported by the use of highly conservative statistical thresholds with correction for multiple comparisons, aiming at uncovering only the most reliable possible activations/deactivations and significant differences.

Few evidences on task fMRI in ET are available so far. Indeed, only seven studies have hitherto investigated ET using fMRI with a motor task and their findings are largely not comparable among each other neither to the results from this study due to inconsistencies in patient selection, differences in the acquisition scheme (mainly paradigms and tasks) and analysis methods^19,30–32,34,44,45^. Our study appears partly comparable with only two previous fMRI studies sharing nearly identical motor tasks^30,44^. Bucher et al. performed quite a pioneering study on a 1.5T MRI scanner and applied a different analysis approach, focusing on the temporal correlation between the time course of each voxel and the stimulus protocol rather than using GLM analyses, as widely done nowadays. In contrast to our findings, Bucher et al.^30^ reported a significantly increased activation in the cerebellar hemispheres and ipsilateral red nucleus during tremor in ET patients compared to mimicked tremor in HC. No between-group differences were found in the activation of primary sensory-motor areas, globus pallidus, thalamus, and dentate nucleus. Likewise our study, Broersma et al.^44^ used a block-related paradigm and similar tasks, although they added EMG recordings which were further used in their analyses to infer both block-related and tremor-related fMRI activations. Broersma et al.^44^ detected block-related activations in the homolateral cerebellum, namely right lobule V, VI, and VIIIa, which further extend to right lobule VIIIb and the contralateral cerebellar hemisphere (left lobule V, VI, VIIb, and IX) when tremor-related analysis was performed. In our study, we found similar clusters of activations in the homolateral cerebellar hemisphere, although their extension was lower possibly due to the more restrictive statistical threshold we used in our analysis (*p* < 0.05 FWE-corrected vs *p* < 0.001 uncorrected). On the contrary, we mainly found deactivated regions in the contralateral cerebellar hemisphere.

In the present study, concerning the activated areas in task 1, ET patients showed stronger activations over several frontal areas compared to HC performing the same task or simulating the tremor (task 2). Frontal areas are involved in planning motor action, movement initiation, maintenance, and planning of complex sequences. Moreover, neural activation patterns among these regions are involved in executive functioning and verbal working memory. Some preliminary studies demonstrated abnormally enhanced responses of prefrontal and parietal cortices in ET patients compared to HC while executing attentional and working memory tasks^45,46^. This was also confirmed with rs-fMRI, revealing enhanced functional activity in specific resting state networks, such as the frontoparietal network, which correlated with increased disease severity, disease duration, and reduced cognitive ability^20,29^. Likewise, our results demonstrate the presence of an increased activity in frontal and prefrontal regions in ET patients while performing a tremor-inducing motor task which might require an additional level of attention for them. In other terms, we speculate that ET patients need to exert greater control to maintain their right arm outstretched in a fixed position due to presence of postural tremor compared to HC. This is also in agreement with a recent study on Parkinson’s disease and ET assessing the cortical activity patterns during bimanual tapping with EEG recordings^47^. The authors found in the ET group an increased task related power in frontal areas involved in movement initiation and planning of complex sequences, confirming that ET patients require more attention, focus and control for correctly performing specific hand movements compared to HC.

To the best of our knowledge, previous studies on task fMRI in ET have never reported BOLD signal decreases (deactivations), having focused only on activation patterns. This parallels the broader literature on task fMRI, which rarely reported on BOLD deactivations because of analysis methods which did not highlight them and/or difficult interpretations of their significance. Negative BOLD responses were previously shown to reflect task-related decrease in neural activity compared to the spontaneous activity level at rest^35,36^. However, since statistical areas are obtained from the comparison between an active and a rest condition, it is difficult to determine whether decreased BOLD signals are pointing towards the deactivation of brain areas during the experimental task, as generally considered nowadays, or the activation of the same areas during the rest condition. Interpretations of BOLD deactivations are still controversial, and can be summarized into two main categories:

1. Deactivations might represent a direct hemodynamic consequence (‘blood stealing’) occurring in response to flow changes in adjacent brain areas, since negative BOLD signals have previously been demonstrated to correspond to either local flow decreases^48,49^ or transient redistribution of blood within a neural network^50^;
2. Deactivations could represent an inhibition process (i.e., an active suppression of neural activity in order to minimize the influence of other task-irrelevant neural processes) as revealed by several studies which show a relationship between negative BOLD signals and decreases in neural activity, therefore suggesting that this phenomenon is predominantly neuronal in origin and not driven by vascular steal^51–53^.

Despite controversies, BOLD deactivations have gained increasing interest since their formalization by Raichle et al. almost twenty years ago^48^. In this seminal paper, the authors proposed that the deactivation pattern may resemble a default state of the brain, involving areas whose activities are attenuated and/or suspended during specific and attention-demanding tasks. This peculiar deactivated map, called default-mode network (DMN), involves the posterior cingulate cortex, the medial frontal cortex and the angular gyrus region of the inferior parietal cortex, and has been replicated in numerous studies. However, further studies with cognitively demanding tasks proved that the deactivation patterns only marginally encompass the DMN, extending beyond this specific network on regions that are responsive to changes in task demand, such as the insula^39^. Tasked-related deactivation is thus not limited to a fixed set of specialized regions and can occur in any brain region that is not apparently involved in controlling or processing the specific task^54^. These findings are consistent with our observations which demonstrated for the first time in ET a widespread deactivated network associated with postural tremor, including areas of the DMN, sensory-motor network, cerebellum and deep structures as insula, amygdala and thalamus, further confirming the presence of a disrupted cerebello-thalamo-cortical tremor network. In addition, the widespread deactivation network observed in the present study might reflect the spatial/temporal interaction between the DMN and other task-specific functional areas. When focusing on the cerebellum, a predominance of contralateral fMRI deactivations was found in ET patients. The interpretation of the present findings is controversial and remains purely speculative in view of the controversy on the significance of fMRI deactivations in previous literature and the lack of previous studies reporting fMRI deactivations in ET. The critical role of the cerebellum in the control of voluntary limb movements and movement-related sensory data acquisition has previously been established^55^. In addition, great emphasis has been given to cerebellar dysfunction/pathology in ET^4,6–8^. Overall, our findings on reduced cerebellar activations in ET patients compared to HC would support an ET-related impairment of cerebellar circuitry. In particular, during task 1, we found a significantly lower activation of the homolateral cerebellar hemisphere in ET compared to HC. We therefore speculate that the predominant contralateral deactivations in ET patients may represent an attempt of their underfunctioning homolateral hemisphere to gather its remaining capacity to fulfil its physiological role, i.e. controlling a task triggering tremor and therefore initiating acquisition of additional tremor-related sensory data. This is likely to represent a higher demanding sensorimotor processing compared to that required in HC. Following our speculation and considering the possible significance of negative BOLD responses, we might hypothesize that deactivations represent a direct hemodynamic consequence (‘blood stealing’) or an active suppression of neural activity possibly occurring to prioritize/maximize task-relevant neural processes in the homolateral cerebellar hemisphere.

Overall, deactivations can unveil relevant aspects of tremor generation and should be explored in future research on larger cohorts of patients with ET, as this study reveals some extent of overlap between deactivated areas and previous pathological findings showing neurodegeneration in the same areas^42^. As additional proof of the altered functionality of these areas, some of them within the motor system showed altered connectivity patterns on previous rs-fMRI studies, which mainly revealed decreased connectivity within the cerebellum and an opposite trend over the pre/postcentral gyri, anterior cingulate and supplementary motor areas^20,56,57^.

Finally, the assessment of a possible linear relationship between tremor scales and BOLD (de)activations (expressed as *z-*statistic values) revealed significant negative correlations over several voxels and regions. When focusing on the activated areas resulting from the GLM analysis, ET patients with low tremor scores showed higher activations, whereas for increased tremor scores they showed a tendency to lower activation or even a shift to deactivation, with highly negative *z*-values for those patients with severe tremor. Conversely, when considering the deactivated areas, almost all patients revealed no activation and negative *z-*values which tended to become higher in absolute value as the tremor severity and body distribution increased. Therefore, the higher the severity and number of affected body segments the strongest the deactivation. These findings hold for all the three TRS subscores considered in the linear regression analysis, thus highlighting the different patterns of activations/deactivations at different level of tremor severity and body distribution and that more severe patients present a more disrupted tremor network.

In order to verify whether the mild inter-side difference in tremor severity might have determined differences in the intensity of fMRI modulations, we performed a correlation analysis between the intensity of fMRI (de)activations and the Fahn–Tolosa–Marin TRS, part A scores for both the right and left upper limb separately. In addition, we performed a correlation analysis between the intensity of fMRI (de)activations and the normalized tremor asymmetry index which was calculated as reported elsewhere^58^. The first analysis confirms significantly negative correlations for both sides as found for the overall TRS-A score. In the second analysis, we did not find any significant correlation. These observations suggest that the mild inter-side difference in tremor severity is unlikely to have determined relevant differences in the intensity of fMRI (de)activations. The severity of tremor itself rather than its asymmetry appeared therefore related to fMRI modulations.

We acknowledge that this study has several limitations. First, the small sample size of the study cohort may have limited statistical power to identify less robust effects. However, compared to previous studies, we enrolled a highly homogeneous cohort of patients with ET diagnosed according to the 2018 strict consensus criteria. Second, our ET cohort has later onset and short disease duration than in previous studies of advanced neuroimaging in ET and this needs to be considered when comparing findings. However, our study seems to capture the later peak of the bimodal distribution of ET age of onset which is well established in previous literature^59,60^. Third, we did not use an MRI-compatible electromyography to determine the frequency of simulated tremor in HC, although the performance of the movement was monitored by an operator throughout the entire acquisition. Finally, considering again the relatively small number of patients studied, the linear regression analyses need to be replicated using a larger sample to further confirm our findings of an inverse relationship between tremor scales and BOLD values, which however already reached a very high statistical significance.

In conclusion, application of combined VBM and task fMRI to a homogeneous cohort of patients with ET demonstrated the involvement of cerebellar areas and clear patterns of widespread deactivations in tasks triggering tremor, which encompassed both cortical and subcortical regions. Limited temporal resolution of BOLD-based fMRI prevents the identification of primary tremor generators or oscillatory mechanisms. However, taken together, our findings provide support for the key role of the cerebellum in the pathophysiology of ET and for the presence of a loop involved in tremor generation, encompassing known structures as cerebellar hemispheres, cortical motor areas, thalamus and pallidum.

## Materials and methods

### Participants

Ten ET patients (6 males, 69.4 ± 8.9 years) were consecutively enrolled at the Movement Disorders Centre of the University Hospital of Verona, Italy. ET was diagnosed according to the 2018 consensus criteria^1^. Exclusion criteria were: presence of moderate to severe head tremor (score > 2 on the Tremor Research Group Essential Tremor Rating Assessment Scale)^61^; presence of rest tremor and/or other neurological signs; presence of cognitive impairment (Mini Mental State Examination $\le \hspace{0.17em}$24); history of neurological or psychiatric diseases; current or past exposure to tremorgenic drugs; contraindication for MRI. In ET patients who were taking medications for tremor, drugs were stopped at least 72 h before the study session, which consisted of clinical assessment and MRI scanning performed on the same day. Ten age-matched healthy subjects (5 males, 67.7 ± 7.8 years) with no history of neurological or psychiatric diseases and unremarkable neurological assessment were recruited as controls.

Participants were asked to avoid caffeine, theine, and alcohol intake over 12 h preceding the study session. ET patients and HC gave their written informed consent to participate in the study, which was approved by the local Ethics Committee for Clinical Sperimentation (CESC) of Verona and Rovigo (no. 1482CESC) and conducted in accordance with the Declaration of Helsinki (2008).

### Clinical assessment

Age of onset, disease duration, family history of ET or other neurological diseases, and effect of alcohol on tremor were collected. ET patients underwent a throughout neurological assessment by a movement disorder specialist and tremor was rated using the Fahn–Tolosa–Marin TRS^62^. TRS includes three parts. Part A was used to assess tremor amplitude in different body sites (items 1–9, score: 0–84), part B to examine handwriting, drawing, and pouring (items 10–14, score: 0–36), and part C to evaluate the burden of tremor on activities of daily living (items 15–21, score: 0–28)^62^. Assessment of depression and anxiety in both ET patients and HC was obtained by the self-administered questionnaires BDI-II (score: 0–63, higher score indicates more severe depression) and STAI Y2 for trait anxiety (score: 20–80, higher score indicates greater trait anxiety)^63,64^.

As for demographic and clinical data, absolute and relative frequencies were tested by Fisher’s Exact test. For continuous data, descriptive statistics are reported in terms of means and standard deviations and comparisons between groups were performed using non-parametric Mann–Whitney U test due to the small sample size (*p* < 0.05). All statistical analyses were carried out using SPSS® Statistics (version 21.0).

### Experimental protocol and image acquisition

ET patients and HC underwent MRI scanning on a 3T system (Philips Achieva, Philips Medical Systems, Netherlands) equipped with an 8-channel head coil. They laid supine inside the bore of the scanner with their arms along the body and forearms pronated. The head was stabilized with adjustable padded restraints on both sides. Subjects were instructed to remain as still as possible and to keep their eyes closed throughout the experiment.

In ET patients, block-designed BOLD fMRI was performed during the execution of a motor task, i.e. maintaining the right arm outstretched leading to the appearance of tremor (task 1). Conversely, HC were scanned during two separate block-designed paradigms: (1) while maintaining the right arm outstretched, as in ET patients (task 1); (2) while mimicking a tremor through flexion–extension of the right wrist at the highest frequency they could reach with the arm outstretched (task 2). As previously reported^30^, the mimicked tremor task in controls should be considered as the optimal voluntary motor task for comparisons with the postural tremor in ET patients. Participants were externally cued to switch from rest (arms along the body) to active task and back to rest by the auditory inputs “start” and “stop” presented via headphones and monitored in real-time by one evaluator. To ensure that tasks were performed as accurately as possible, all participants were trained for several minutes before undergoing MRI and monitored by an examiner throughout the whole acquisition.

Task fMRI data were acquired using 2D gradient-echo echo-planar imaging and the following parameters: TR/TE = 2000/30 ms; 36 slices, 3 × 3 × 4 mm^3^, no slice gap, FOV = 192 × 192 mm^2^; flip angle = 75°. Each block-designed paradigm consisted of thirteen 20 s cycles of rest alternated with thirteen 20 s cycles of task, for a total of 260 volumes. A 3D T1-weighted turbo field echo scan was finally acquired for each subject (TR/TE = 8.16/3.73 ms; 180 slices, 1 × 1 × 1 mm^3^, no slice gap, FOV = 256 × 256 mm^2^; flip angle = 8°).

### VBM analysis

VBM analysis was performed using the optimised FSL-VBM protocol available in FSL 5.0.9 (https://fsl.fmrib.ox.ac.uk/fsl/fslwiki/)^65^. T1-weighted images were brain-extracted (BET) and segmented into GM, WM and cerebrospinal fluid (CSF) using the FMRIB’s Automated Segmentation Tool FAST. GM maps were affine-registered to the GM ICBM-152 template, concatenated and averaged across study subjects. This averaged image was flipped along the x-axis and the two mirror images re-averaged to obtain a first study-specific GM template. Individual GM images were subsequently non-linearly re-registered to this template, concatenated, averaged, and flipped along the x-axis. Both mirror images were finally averaged to create the symmetric, study-specific GM template (2 × 2 × 2mm^3^, MNI space). Afterwards, each individual GM image was non-linearly registered to the study template, modulated to compensate for the contraction/enlargement due to the non-linear component of the transformation and smoothed (Gaussian kernel, sigma = 3 mm). A permutation-based non-parametric inference (5000 permutations) with the threshold-free cluster enhancement (TFCE) option was finally performed to statistically compare the GM images of the two groups. The significance level was set at *p* < 0.05 and corrected for multiple comparisons (FWE).

### fMRI data analysis and statistical comparisons

Functional data were first minimally preprocessed using FSL 5.0.9. The pipeline included head motion correction (MCFLIRT), non-brain tissue removal (BET), spatial smoothing (isotropic Gaussian filter with a full-width-at-half-maximum of 6 mm^3^), and high-pass temporal filtering for removing slow drifts (0.01 Hz). Participant head motion profiles (three translations and three rotations) were statistically compared across conditions in order to test for possible differences in one or more motion parameters (non-parametric Wilcoxon tests, *p* < 0.05). Preprocessed data were registered to the T1-weighted image by applying a linear registration (FLIRT) with boundary-based registration (BBR) cost function^66^. Each T1-weighted image was then registered to the 2-mm MNI standard space using a non-linear method (FNIRT). Finally, the joint BOLD/T1-weighted and T1-weighted/MNI transformation parameters were used to spatially normalize the functional data.

To identify activated/deactivated voxels at the single-subject level, a GLM analysis was performed^67^. The stimulus-related regressor in the design matrix was assumed to be a vector describing the BOLD effect changes for the motor tasks. This was obtained by convolving the boxcar waveform, representing the experimental protocol, with a hemodynamic response function modelled by a canonical double-gamma function. Moreover, in order to account for movement-related artefacts and other non-neuronal fluctuations not completely eliminated by preprocessing, the six motion parameters from MCFLIRT were included as nuisance confounds in the design matrix, along with the average WM and CSF signals. These two regressors were calculated as the average of time courses within the corresponding tissue masks generated by segmenting the spatially normalized structural images (FAST) and thresholding the tissue probability maps at 0.9. Moreover, we took the probability of GM volume as further voxelwise covariate.

For each subject, a t-test was applied to create a *z*-statistic map for each dataset. These lower-level statistical maps were fed into the higher-level group analysis (FEAT). For task 1, a fixed-effects (FE) analysis was applied to estimate the group results in ET patients and HC, highlighting both the activated areas related to the task, and the deactivations. The high-level statistical maps were thresholded using clusters determined by Z > 6 (activations) and Z < − 6 (deactivations) plus a (corrected) cluster significance threshold of *p* < 0.05. A separate FE analysis was applied to the HC data for task 2 (same statistical threshold as for task 1).

Three statistical analyses were finally performed to compare the identified brain areas: (1) *between-group analysis* (task 1 in ET patients vs HC); (2) *between-group between-task analysis* (task 1 in ET patients vs task 2 in HC); (3) *within-group analysis* (task 1 vs task 2 in HC). All these statistical maps were thresholded using clusters determined by Z > 4 (activations) and Z < − 4 (deactivations) plus a (corrected) cluster significance threshold of *p* < 0.05.

### fMRI linear regression analysis with TRS

Regression analysis was carried out (fsl_glm) to voxelwise assess whether a linear relationship exists between TRS scores and BOLD (de)activations in ET patients. In particular, part A, A + B and A + B + C (total) of the TRS were separately considered as clinical variables in a linear regression. For fMRI, individual *z-*statistic maps resulting from lower-level single-subject analysis were used as representative values of BOLD (de)activations. For each analysis, resulting *p*-values were corrected for multiple comparisons (FDR) and only corrected *p*-values < 0.05 were considered as statistically significant. These statistical maps were finally masked using (de)activation clusters resulting from the patient group analysis, in order to restrict the assessment of the clinical/imaging link to the areas elicited by task 1.

## Supplementary information

Supplementary Information.

## Data Availability

Datasets for this study are available from the corresponding authors upon reasonable request.
