# Supplementary material for: Voxel-based morphometry and task functional magnetic resonance imaging in essential tremor: evidence for a disrupted brain network
Source: Sci Rep. 2020 Sep 15;10:15061. doi: 10.1038/s41598-020-69514-w (PMC7493988; doi:10.1038/s41598-020-69514-w)

**Voxel-based morphometry and task functional magnetic resonance imaging in essential tremor: evidence for a disrupted brain network**

Ilaria Boscolo Galazzo^1§^*, Francesca Magrinelli^2§^*, Francesca Benedetta Pizzini^3^,

Silvia Francesca Storti^1^, Federica Agosta^4^, Massimo Filippi^4^, Angela Marotta^2^,

Giancarlo Mansueto^3^, Gloria Menegaz^1^, Michele Tinazzi^2^

^1^ Department of Computer Science, University of Verona, Verona, Italy

^2^ Department of Neurosciences, Biomedicine and Movement Sciences, University of Verona, Verona, Italy

^3^ Department of Diagnostics and Pathology, University of Verona, Verona, Italy

^4^ Neuroimaging Research Unit, Institute of Experimental Neurology, Division of Neuroscience, San Raffaele Scientific Institute, Vita-Salute San Raffaele University, Milan, Italy

^§^ These authors equally contributed as first author to this work.

**Table S1 –** Participant head motion parameters during task 1 and task 2 for healthy controls (HC) and during task 1 for patients with essential tremor (ET). The six motion parameters (translation: x, y and z in mm; rotation: pitch, roll and yaw in degrees) were obtained from head correction for each participant and are reported as mean ± standard deviation values across group. Values were statistically compared, revealing no statistically significant changes across different groups/tasks (*p* > 0.05).

|  |  |  |  |  |  |  |  |
| --- | --- | --- | --- | --- | --- | --- | --- |
|  | **HC** | | **ET** |  | **Comparisons (*p*-values)** | | |
|  | **Task 1** | **Task 2** | **Task 1** |  | **Task 1 HC vs Task 1 ET** | **Task 2 HC vs Task 1 ET** | **Task 1 HC vs Task 2 HC** |
|  |  |  |  |  |  |  |  |
| **x** | -0.0045 ± 0.1555 | -0.0050 ± 0.1786 | 0.0076 ± 0.1962 |  | 0.3256 | 0.6232 | 0.6776 |
| **y** | 0.0153 ± 0.0840 | -0.0302 ± 0.1040 | 0.0414 ± 0.1105 |  | 0.7337 | 0.4274 | 0.7337 |
| **z** | -0.1061 ± 0.1686 | -0.0206 ± 0.2470 | -0.0620 ± 0.1861 |  | 0.1405 | 0.4274 | 0.4727 |
| **Pitch** | 0.0013 ± 0.0024 | 0.0017 ± 0.0061 | -0.0027 ± 0.0063 |  | 0.5202 | 0.2119 | 0.7335 |
| **Roll** | 0.0002 ± 0.0011 | -0.0001 ± 0.0016 | 0.0006 ± 0.0018 |  | 0.7048 | 0.4485 | 0.7047 |
| **Yaw** | 0.0004 ± 0.0014 | 0.0003 ± 0.0010 | -0.0007 ± 0.0014 |  | 0.1499 | 0.1610 | 1.0 |

Abbreviations. ET = patients with essential tremor; HC = healthy controls.

**Table S2 -** Group GLM results showing brain regions exhibiting significant activation during task 1 (patients with essential tremor [ET] and healthy controls [HC]) and task 2 (HC only) compared with the rest condition.

| **TASK 1 - ACTIVATIONS** | **HC** | | | | | |  |
| --- | --- | --- | --- | --- | --- | --- | --- |
|  |  |  |  |  |  |  |  |
|  | **ROI** | **Voxels** | **MAX *Z-*statistic** | **MAX X (mm)** | **MAX Y (mm)** | **MAX Z (mm)** |  |
|  | CER.r | 5330 | 11.7 | 43 | 30 | 24 |  |
|  | TP.r/HIPP.r | 1200 | 6.98 | 23 | 66 | 22 |  |
|  | CGa.r/CGa.l | 813 | 6.81 | 49 | 84 | 36 |  |
|  | FP.r | 770 | 6.65 | 27 | 85 | 34 |  |
|  | FP.l | 644 | 7.21 | 54 | 91 | 43 |  |
|  | PRG.l/POG.l | 535 | 9.37 | 60 | 45 | 63 |  |
|  | Caud.r/Caud.l | 493 | 7.79 | 40 | 63 | 43 |  |
|  | Put.l | 249 | 6.63 | 59 | 58 | 36 |  |
|  | Put.r/INS.r | 228 | 6.11 | 32 | 63 | 36 |  |
|  | SMA | 205 | 6.01 | 48 | 52 | 63 |  |
|  | THL.r/THL.l | 202 | 6.58 | 44 | 55 | 37 |  |
|  | CER.l | 201 | 6.17 | 66 | 42 | 18 |  |
|  | **ET** | | | | | |  |
|  |  |  |  |  |  |  |  |
|  | **ROI** | **Voxels** | **MAX *Z-*statistic** | **MAX X (mm)** | **MAX Y (mm)** | **MAX Z (mm)** |  |
|  | FP.r/FP.l | 5652 | 8.04 | 56 | 91 | 39 |  |
|  | PRG.r/MFG.r | 1446 | 9.78 | 16 | 67 | 46 |  |
|  | CER.r | 1340 | 11.3 | 42 | 31 | 27 |  |
|  | Caud.r/Caud.l | 1080 | 9.26 | 37 | 75 | 34 |  |
|  | TP.r/HIPP.r | 678 | 6.42 | 23 | 68 | 22 |  |
|  | CGa.r/CGa.l | 650 | 7.11 | 47 | 81 | 39 |  |
|  | PRG.l/POG.l | 455 | 9.94 | 61 | 58 | 60 |  |
|  | POC.r | 324 | 6.92 | 18 | 51 | 44 |  |
|  |  |  |  |  |  |  |  |
| **TASK 2 - ACTIVATIONS** | **HC** | | | | | |  |
|  |  |  |  |  |  |  |  |
|  | **ROI** | **Voxels** | **MAX *Z-*statistic** | **MAX X (mm)** | **MAX Y (mm)** | **MAX Z (mm)** |  |
|  | CER.l/CER.r | 8567 | 29.8 | 35 | 36 | 24 |  |
|  | PRG.l/POG.l | 3522 | 27.2 | 66 | 49 | 60 |  |
|  | THL.r/THL.l/Caud.r/Caud.l | 818 | 14.9 | 34 | 53 | 44 |  |
|  | MFG.l/IFG.l | 637 | 14.5 | 74 | 65 | 35 |  |
|  | PRG.r/POG.r/INS.r | 629 | 14.8 | 14 | 69 | 43 |  |
|  | SMA | 589 | 15.3 | 48 | 51 | 61 |  |

Voxels = number of voxels in each significant cluster; MAX *Z-*statistic = value of the maximum z-statistic within the cluster; MAX X/Y/Z (mm) = location of the maximum intensity voxel, given as spatial coordinate values in standard space (mm). For all clusters, the corresponding *p-*values are FWE-corrected and < 0.05. For ease of reading, only significant clusters with at least 200 voxels are reported for the GLM activations.

Abbreviations. Caud = caudate; CER = cerebellum; CGa = anterior cingulate gyrus; ET = patients with essential tremor; FP = frontal pole; HC = healthy controls; HIPP = hippocampus; IFG = inferior frontal gyrus; INS = insular cortex; l = left; MFG = middle frontal gyrus; POC = parietal opercular cortex; POG = postcentral gyrus; PRG = precentral gyrus; Put = putamen; r = right; ROI = region of interest; SMA = supplementary motor areas; THL = thalamus; TP = temporal pole.

**Table S3 -** Group GLM results showing brain regions exhibiting significant deactivation during task 1 (patients with essential tremor [ET] and healthy controls [HC]) and task 2 (HC only) compared with the rest condition.

| **TASK 1 - DEACTIVATIONS** | **HC** | | | | | | |
| --- | --- | --- | --- | --- | --- | --- | --- |
|  | **ROI** | **Voxels** | **MAX *Z-*statistic** | **MAX X (mm)** | **MAX Y (mm)** | **MAX Z (mm)** |  |
|  | FP.l/FP.r | 1249 | -8.91 | 70 | 82 | 45 |  |
|  | OP.r | 1152 | -8.34 | 39 | 11 | 38 |  |
|  | CER.l | 1126 | -9.39 | 54 | 18 | 20 |  |
|  | CER.r | 661 | -9.06 | 32 | 18 | 22 |  |
|  | OP.l | 400 | -7.47 | 47 | 11 | 35 |  |
|  | SPL.r | 235 | -6.86 | 27 | 40 | 66 |  |
|  | SPL.l | 203 | -6.26 | 54 | 33 | 68 |  |
|  | **ET** | | | | | | |
|  | **ROI** | **Voxels** | **MAX *Z-*statistic** | **MAX X (mm)** | **MAX Y (mm)** | **MAX Z (mm)** |  |
|  | POG.r/POG.l/PRG.r/ PRG.l/  SMA/LOC.r/LOC.l/THL.r/  THL.l/Put.r/Put.l/CGp.r/CGp.l/  PAC | 17910 | -9.51 | 62 | 41 | 54 |  |
|  | CER.l | 8520 | -15.4 | 48 | 19 | 18 |  |
|  | CER.r | 1715 | -11.4 | 35 | 18 | 17 |  |
|  | FP.r | 252 | -6.73 | 25 | 88 | 49 |  |
|  | INS.r/AMY.r | 224 | -7.36 | 32 | 60 | 28 |  |
|  | LgG.r | 203 | -8.56 | 32 | 39 | 35 |  |
|  |  |  |  |  |  |  |  |
| **TASK 2 - DEACTIVATIONS** | **HC** | | | | | | |
|  | **ROI** | **Voxels** | **MAX *Z-*statistic** | **MAX X (mm)** | **MAX Y (mm)** | **MAX Z (mm)** |  |
|  | MFG.l/FP.l | 523 | -13.5 | 64 | 90 | 46 |  |
|  | OP.r | 273 | -12.9 | 32 | 16 | 28 |  |
|  | CGp.l | 207 | -9.05 | 46 | 39 | 51 |  |

Voxels = number of voxels in each significant cluster; MAX *Z-*statistic = value of the maximum z-statistic within the cluster; MAX X/Y/Z (mm) = location of the maximum intensity voxel, given as spatial coordinate values in standard space (mm). For all clusters, the corresponding *p-*values are FWE-corrected and < 0.05. For ease of reading, only significant clusters with at least 200 voxels are reported for GLM deactivations.

Abbreviations. AMY = amygdala; CER = cerebellum; CGp = posterior cingulate gyrus; ET = patients with essential tremor; FP = frontal pole; HC = healthy controls; INS = insular cortex; l = left; LgG = lingual gyrus; LOC = lateral occipital cortex; MFG = middle frontal gyrus; OP = occipital pole; PAC = paracingulate gyrus; POG = postcentral gyrus; PRG = precentral gyrus; Put = putamen; r = right; ROI = region of interest; SMA = supplementary motor areas; SPL = superior parietal lobule; THL = thalamus.

**Table S4 -** Within-group comparison of activated and deactivated brain regions during outstretching of the right arm (task 1) and mimicked tremor (task 2) in healthy controls.

| **ACTIVATIONS** | **TASK 1 > TASK 2** | | | | | |  |
| --- | --- | --- | --- | --- | --- | --- | --- |
|  |  |  |  |  |  |  |  |
|  | **ROI** | **Voxels** | **MAX *Z-*statistic** | **MAX X (mm)** | **MAX Y (mm)** | **MAX Z (mm)** |  |
|  | TP.r/MTG.r | 216 | 7.01 | 12 | 56 | 30 |  |
|  | PAC | 130 | 6.2 | 47 | 76 | 56 |  |
|  | HIPP.r | 117 | 6.46 | 31 | 61 | 25 |  |
|  | FP.l | 102 | 5.82 | 50 | 88 | 34 |  |
|  | **TASK 1 < TASK 2** | | | | | |  |
|  |  |  |  |  |  |  |  |
|  | **ROI** | **Voxels** | **MAX *Z-*statistic** | **MAX X (mm)** | **MAX Y (mm)** | **MAX Z (mm)** |  |
|  | CER.r | 5009 | 21.1 | 35 | 36 | 24 |  |
|  | PRG.l/POG.l | 2444 | 21.9 | 66 | 49 | 59 |  |
|  | CER.l | 730 | 10.6 | 56 | 31 | 23 |  |
|  | POG.r/POC.r | 304 | 11.6 | 14 | 54 | 44 |  |
|  | SMA | 298 | 9.86 | 43 | 54 | 65 |  |
|  | LOC.l (inf) | 204 | 10 | 70 | 29 | 34 |  |
|  | INS.r | 141 | 8.3 | 29 | 69 | 42 |  |
|  | THL.l | 104 | 7.52 | 45 | 60 | 40 |  |
|  |  |  |  |  |  |  |  |
| **DEACTIVATIONS** | **TASK 1 > TASK 2** | | | | | |  |
|  |  |  |  |  |  |  |  |
|  | **ROI** | **Voxels** | **MAX *Z-*statistic** | **MAX X (mm)** | **MAX Y (mm)** | **MAX Z (mm)** |  |
|  | POG.l | 324 | -17.5 | 69 | 50 | 62 |  |
|  | CER.l | 134 | -7.2 | 59 | 32 | 9 |  |
|  | LOC.l (sup) | 115 | -7.02 | 55 | 32 | 63 |  |
|  | **TASK 1 < TASK 2** | | | | | |  |
|  |  |  |  |  |  |  |  |
|  | **ROI** | **Voxels** | **MAX *Z-*statistic** | **MAX X (mm)** | **MAX Y (mm)** | **MAX Z (mm)** |  |
|  | CGp.r/CGp.l | 234 | -8.23 | 47 | 37 | 48 |  |
|  | LOC.r (inf) | 123 | -8.86 | 24 | 19 | 33 |  |
|  | TP.r | 114 | -9.33 | 18 | 59 | 21 |  |
|  | TP.l | 101 | -6.21 | 72 | 57 | 21 |  |

Voxels = number of voxels in each significant cluster; MAX *Z-*statistic = value of the maximum z-statistic within the cluster; MAX X/Y/Z (mm) = location of the maximum intensity voxel, given as spatial coordinate values in standard space (mm). For all clusters, the corresponding *p-*values are FWE-corrected and < 0.05. For ease of reading, a cluster size of at least 100 voxels was chosen for reporting the significant clusters resulting from the statistical comparison.

Abbreviations. CER = cerebellum; CGp = posterior cingulate gyrus; FP = frontal pole; HIPP = hippocampus; inf = inferior; INS = insular cortex; l = left; LOC = lateral occipital cortex; MTG = middle temporal gyrus; PAC = paracingulate gyrus; POC = parietal opercular cortex; POG = postcentral gyrus; PRG = precentral gyrus; r = right; ROI = region of interest; SMA = supplementary motor areas; sup = superior; THL = thalamus; TP = temporal pole.

**Table S5 –** Significant results derived from linear regression analyses between BOLD activations/deactivations and the Fahn-Tolosa-Marin tremor rating scale (TRS, part A, A+B and Total) in patients with essential tremor.

| **TRS, part A** | | | | | | | |
| --- | --- | --- | --- | --- | --- | --- | --- |
| **ROI** | **Coefficient (beta)** | ***p-*value**  **(FDR-corrected)** | **r-value** | **Voxels** | **MAX X (mm)** | **MAX Y (mm)** | **MAX Z (mm)** |
| **ACTIVATION AREAS** | | | | | | | |
| FP.l | -0.631 | 0.002 | -0.776 | 1040 | 58 | 93 | 33 |
| IFG.r/MFG.r | -0.833 | 0.00001 | -0.923 | 941 | 15 | 68 | 40 |
| CGa.r | -0.745 | 0.00001 | -0.879 | 677 | 45 | 81 | 35 |
| FP.r | -0.499 | 0.008 | -0.731 | 329 | 36 | 86 | 32 |
| SFG.r/PAC | -0.756 | 0.004 | -0.774 | 226 | 48 | 87 | 35 |
| PRG.l/POG.l | -0.844 | 0.00001 | -0.898 | 188 | 62 | 51 | 71 |
| POC.r | -0.541 | 0.00001 | -0.644 | 143 | 22 | 49 | 44 |
| TP.r/ITG.r/MTG.r | -0.626 | 0.004 | -0.775 | 119 | 19 | 64 | 17 |
| SGa.r | -0.415 | 0.001 | -0.749 | 119 | 11 | 44 | 48 |
| **DEACTIVATION AREAS** | | | | | | | |
| Caud.l/HIPP.l | -0.484 | 0.002 | -0.697 | 144 | 57 | 59 | 21 |
| CGp.r/PRG.r | -0.416 | 0.001 | -0.861 | 126 | 43 | 45 | 59 |
| POG.l/PRG.l | -0.586 | 0.00001 | -0.891 | 107 | 66 | 43 | 61 |
| **TRS, part A+B** | | | | | | | |
| **ACTIVATION AREAS** | | | | | | | |
| CGa.l/SFG.l/FP.l | -0.317 | 0.00001 | -0.918 | 2921 | 47 | 69 | 35 |
| CGa.r/SFG.r/FP.r | -0.302 | 0.001 | -0.859 | 1063 | 43 | 72 | 30 |
| IFG.r/MFG.r | -0.388 | 0.00001 | -0.892 | 1017 | 18 | 68 | 42 |
| POG.r/PRG.r | -0.183 | 0.00001 | -0.905 | 234 | 16 | 52 | 42 |
| PRG.l/POG.l/SFG.l | -0.288 | 0.003 | -0.803 | 210 | 63 | 59 | 69 |
| **DEACTIVATION AREAS** | | | | | | | |
| Caud.l/Put.l/HIPP.l | -0.256 | 0.00001 | -0.770 | 150 | 58 | 60 | 21 |
| SGa.l/POG.l | -0.232 | 0.001 | -0.727 | 202 | 66 | 41 | 59 |
| CGp.l | -0.149 | 0.00001 | -0.923 | 105 | 43 | 44 | 57 |
| **TRS, Total** | | | | | | | |
| **ACTIVATION AREAS** | | | | | | | |
| SFG.l/SFG.r/FP.l/FP.r/PAC | -0.263 | 0.00001 | -0.716 | 3057 | 49 | 85 | 37 |
| MFG.r/IFG.r/PRG.r | -0.214 | 0.00001 | -0.876 | 635 | 14 | 68 | 41 |
| CER.r | -0.132 | 0.001 | -0.447 | 178 | 38 | 29 | 26 |
| CO.l | -0.202 | 0.001 | -0.840 | 114 | 68 | 50 | 45 |
| PRG.l/POG.l | -0.234 | 0.01 | -0.732 | 105 | 63 | 58 | 60 |
| **DEACTIVATION AREAS** | | | | | | | |
| Cau.l/Put.l/HIPP.l | -0.134 | 0.001 | -0.619 | 123 | 58 | 59 | 21 |
| CGp.l | -0.133 | 0.002 | -0.829 | 115 | 49 | 50 | 56 |
| CGp.r | -0.075 | 0.00001 | -0.655 | 106 | 41 | 45 | 57 |

Coefficient (beta) = regression coefficient, slope; r-value = correlation coefficient; voxels = number of voxels in each significant cluster; MAX X/Y/Z (mm) = location of the maximum intensity voxel, given as spatial coordinate values in standard space (mm).

Abbreviations. Caud = caudate; CER = cerebellum; CGa = anterior cingulate gyrus; CGp = posterior cingulate gyrus; CO = central opercular cortex; FP = frontal pole; FTM-A = Fahn-Tolosa-Marin Tremor Rating Scale Part A (see text); FTM-A+B = Fahn-Tolosa-Marin Tremor Rating Scale Parts A+B (see text); FTM-Total = Fahn-Tolosa-Marin Tremor Rating Scale Parts A+B+C (see text); HIPP = hippocampus; IFG = inferior frontal gyrus; ITG = inferior temporal gyrus; l = left; MFG = middle frontal gyrus; MTG = middle temporal gyrus; PAC = paracingulate gyrus; POC = parietal opercular cortex; POG = postcentral gyrus; PRG = precentral gyrus; Put = putamen; r = right; ROI = region of interest; SFG = superior frontal gyrus; SGa = anterior supramarginal gyrus; TP = temporal pole.

**Figure S1 –** Top: areas of gray matter loss in patients with essential tremor (ET) compared to healthy controls (HC) as revealed by voxel-based morphometry (VBM). The threshold for significant clusters was *p* < 0.05, using family-wise error correction with threshold-free cluster enhancement method. Bottom: activation and deactivation maps derived from group analysis on ET patient data for task 1. Statistical maps are thresholded by using clusters determined by Z > 6 (activations) and Z < -6 (deactivations) with a (corrected) cluster significance threshold of *p* < 0.05.

**
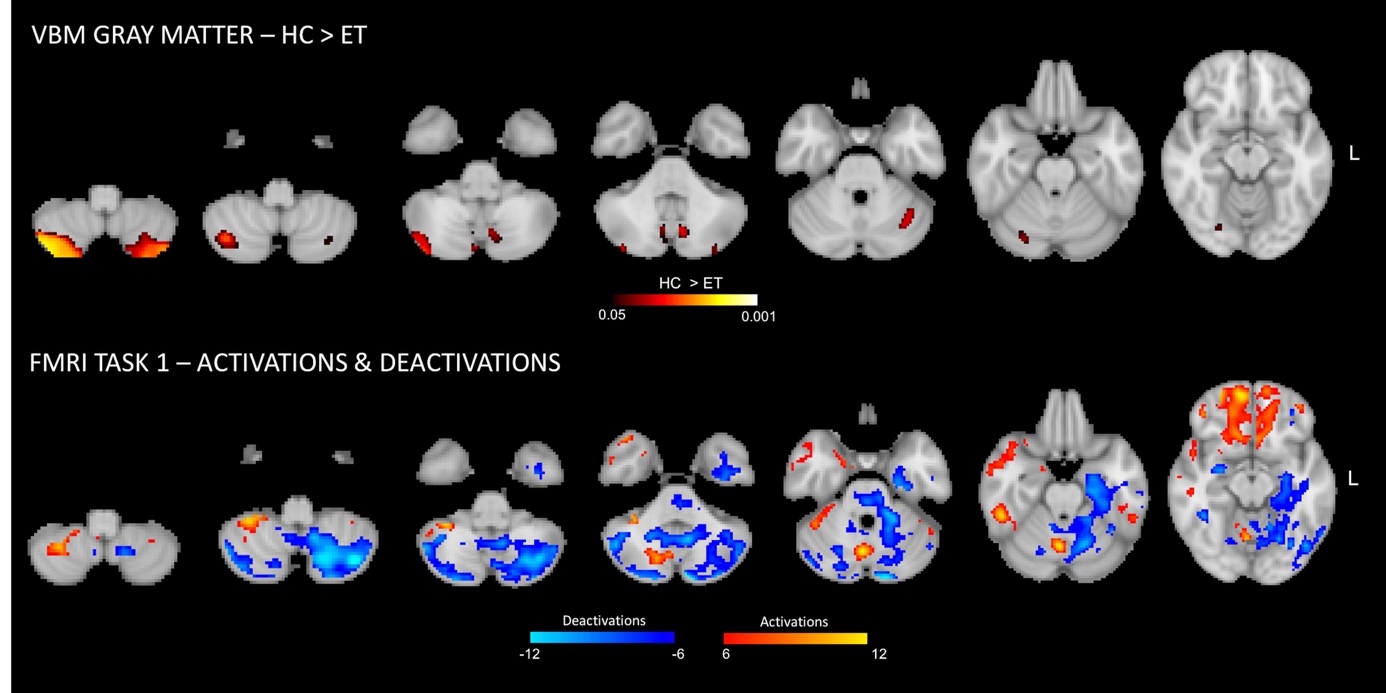
**

**Figure S2 –** Activation and deactivation maps derived from group analyses on healthy controls (HC, task 1 and task 2) and essential tremor (ET) patient data (task 1). Twelve axial slices covering the cerebellar areas are reported. Statistical maps are thresholded by using clusters determined by Z > 6 (activations) and Z < -6 (deactivations) with a (corrected) cluster significance threshold of *p* < 0.05.

**
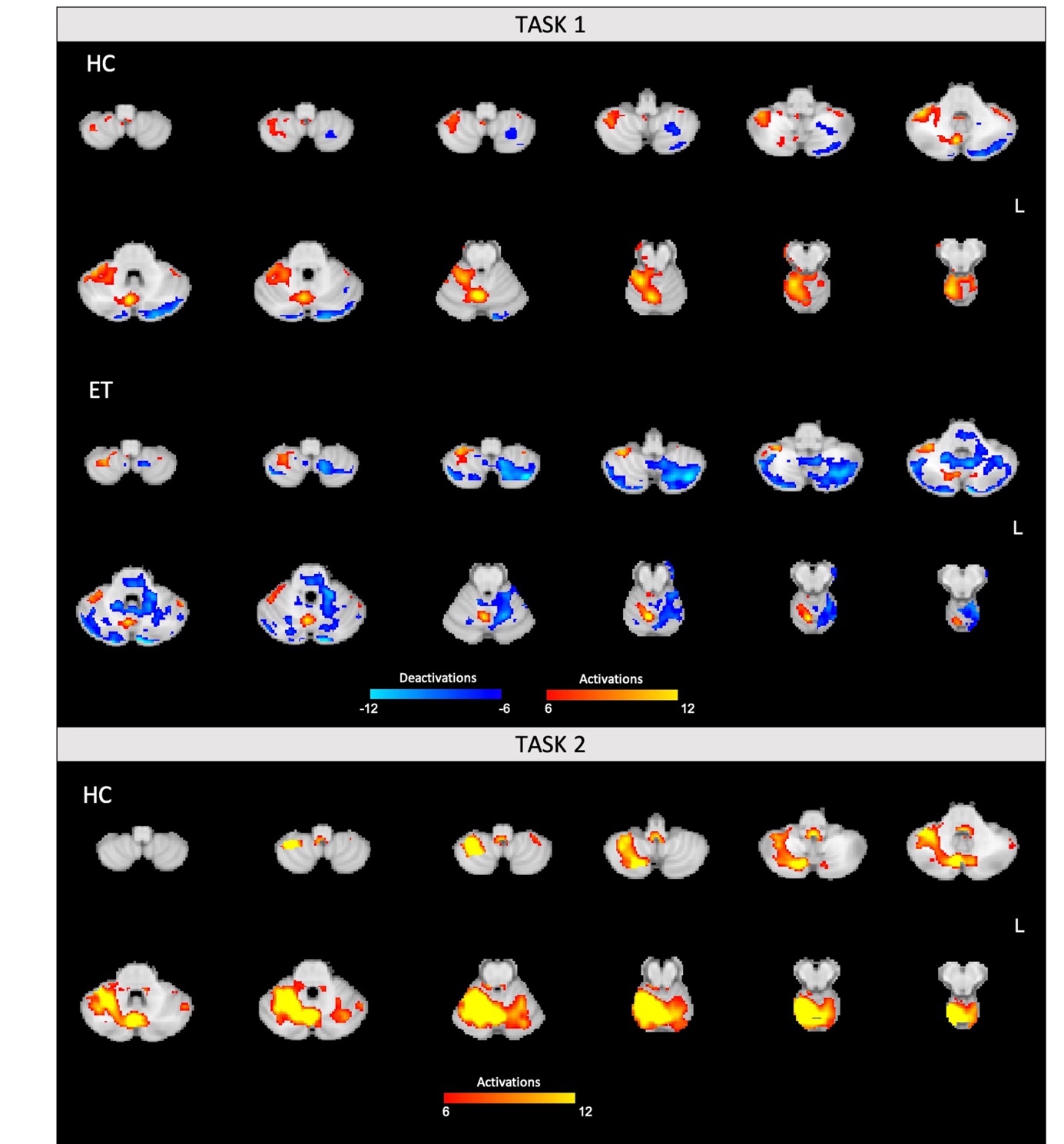
**

**Figure S3 –** Cerebellar areas statistically different between healthy controls (HC) and essential tremor (ET) patients for task 1. Activations and deactivations were separately compared in the statistical analyses, and significant clusters were determined by Z > 4 (activations) and Z < -4 (deactivations) with a (corrected) cluster significance threshold of *p* < 0.05.

**
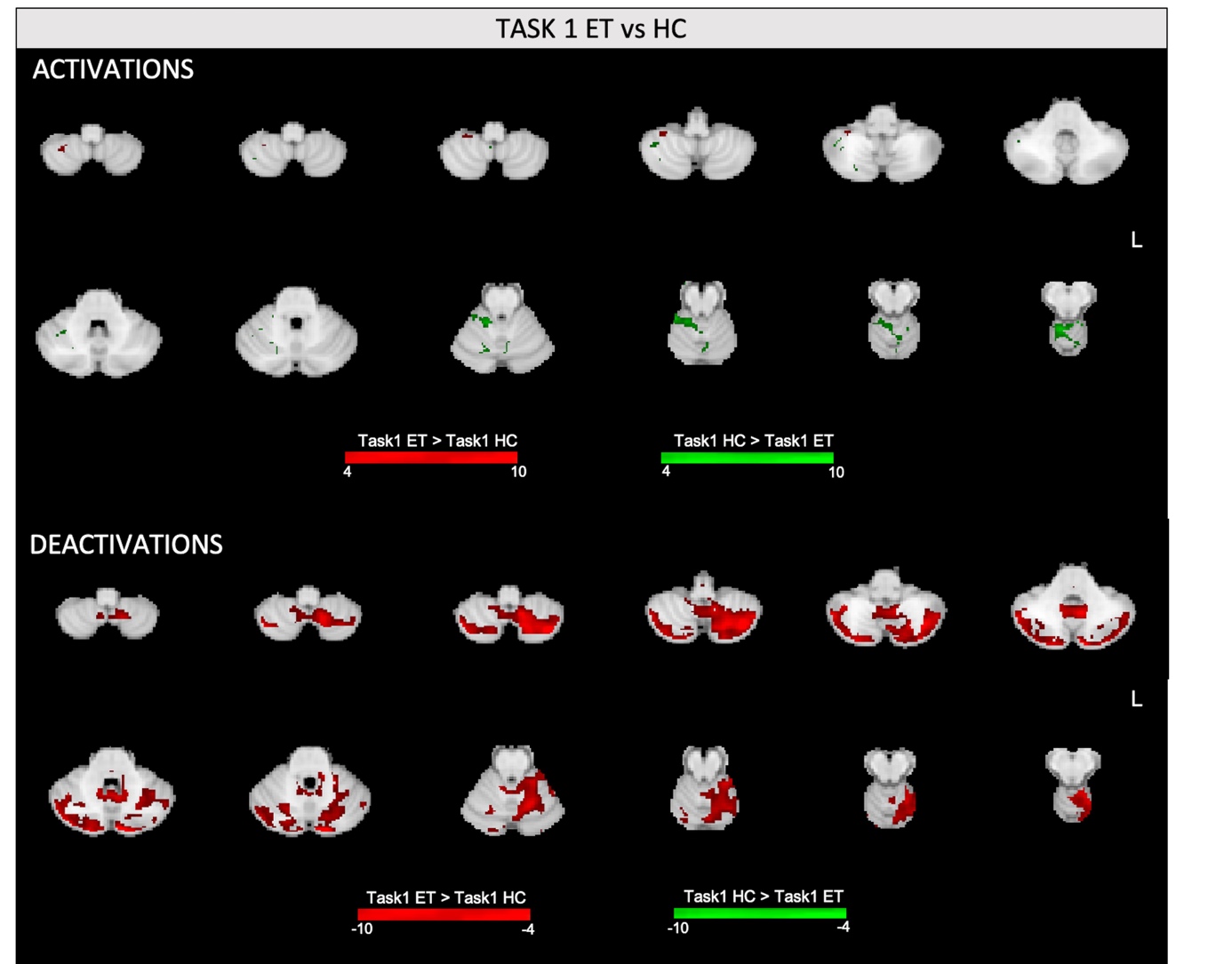
**

**Figure S4 –** Cerebellar areas statistically different between healthy controls (HC, task 2) and essential tremor patients (ET, task 1). Activations and deactivations were separately compared in the statistical analyses, and significant clusters were determined by Z > 4 (activations) and Z < -4 (deactivations) with a (corrected) cluster significance threshold of *p* < 0.05.


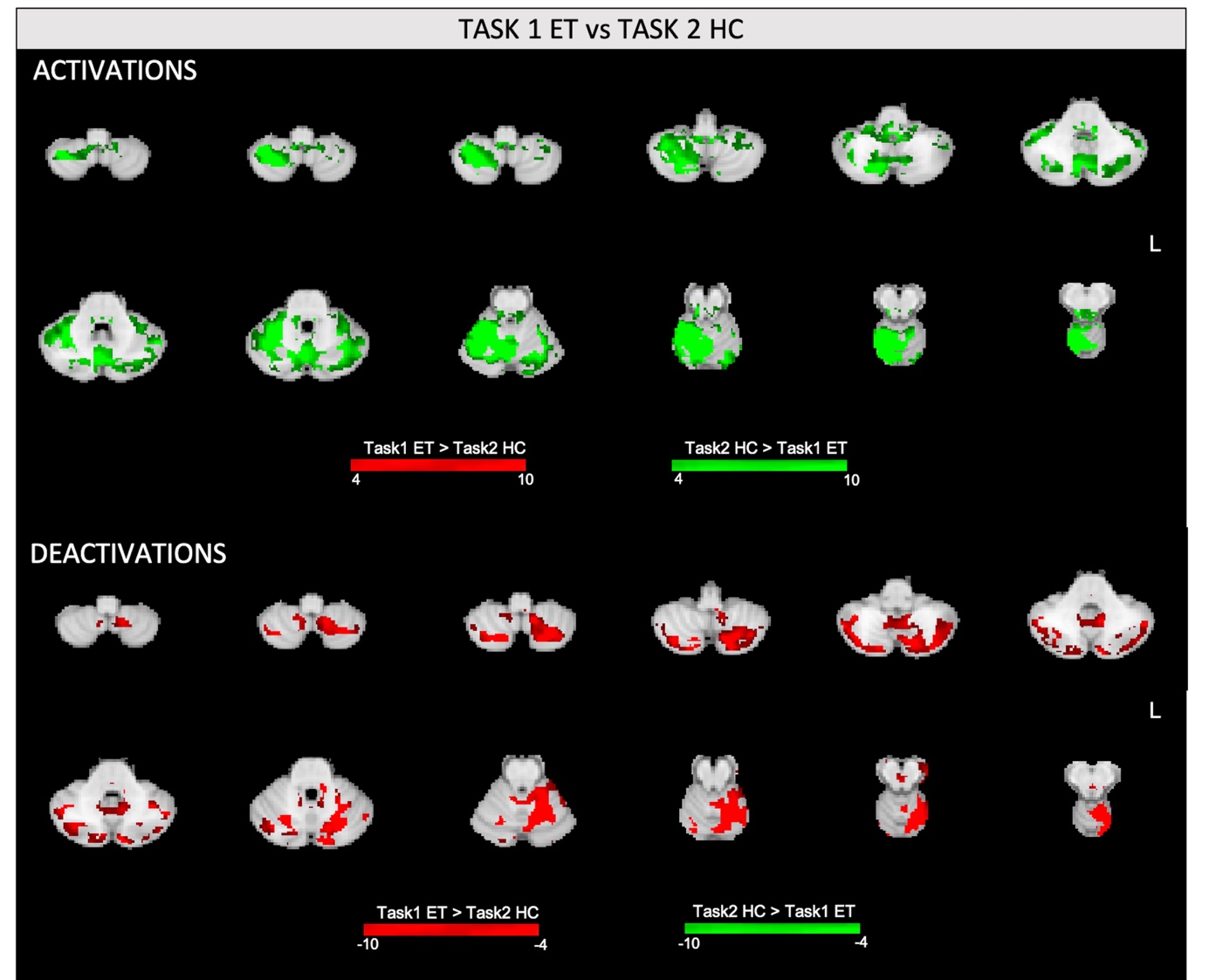


**Figure S5 –** Brain areas statistically different across task 1 and task 2 in healthy controls (HC). Activations and deactivations were separately compared in the statistical analyses, and significant clusters were determined by Z > 4 (activations) and Z < -4 (deactivations) with a (corrected) cluster significance threshold of *p* < 0.05.


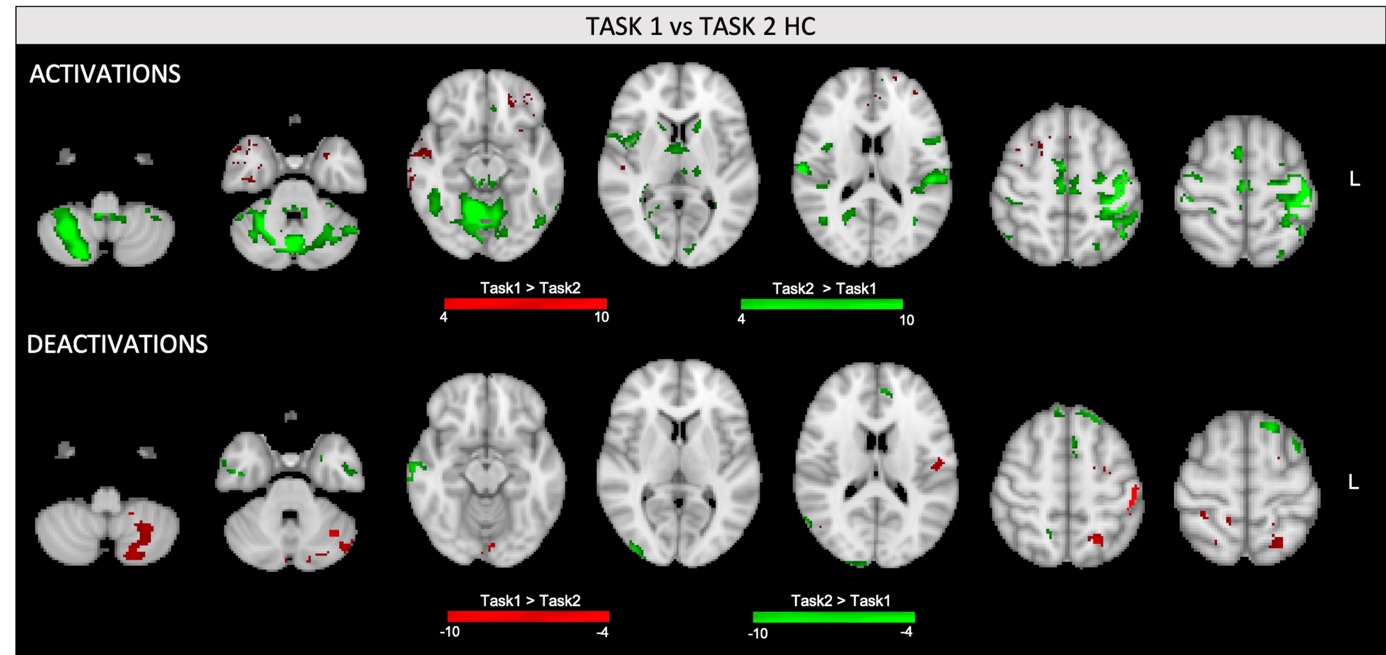


**Figure S6 –** Cerebellar areas statistically different across task 1 and task 2 in healthy controls (HC). Activations and deactivations were separately compared in the statistical analyses, and significant clusters were determined by Z > 4 (activations) and Z < -4 (deactivations) with a (corrected) cluster significance threshold of *p* < 0.05.


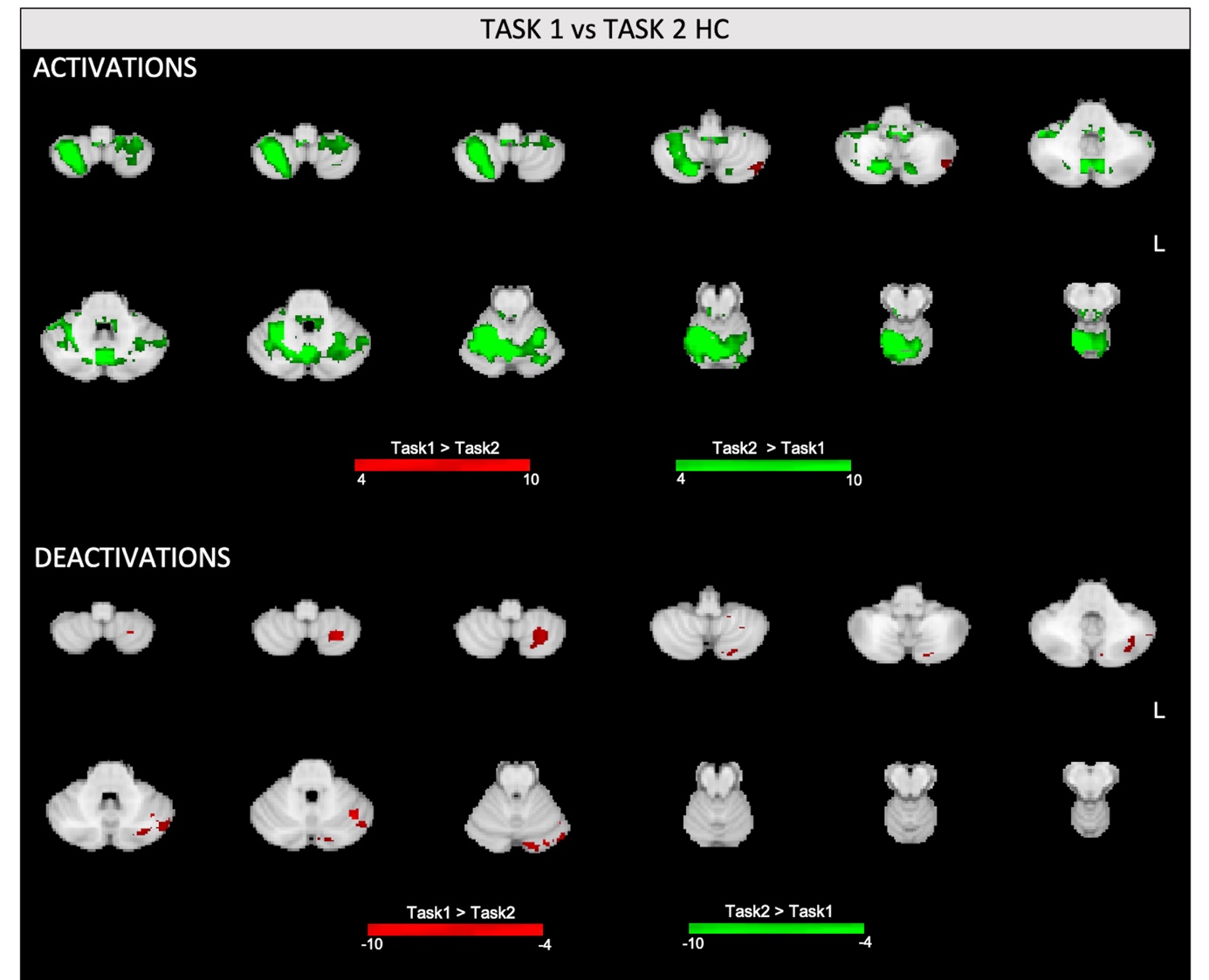

Supplement: Supplementary file 1 — Supplementary Information. [file 41598_2020_69514_MOESM1_ESM.docx]
